# Leaf transcriptome of two highly divergent genotypes of *Urochloa humidicola* (Poaceae), a tropical polyploid forage grass adapted to acidic soils and temporary flooding areas

**DOI:** 10.1186/s12864-016-3270-5

**Published:** 2016-11-11

**Authors:** Bianca Baccili Zanotto Vigna, Fernanda Ancelmo de Oliveira, Guilherme de Toledo-Silva, Carla Cristina da Silva, Cacilda Borges do Valle, Anete Pereira de Souza

**Affiliations:** 1 Embrapa Pecuária Sudeste, São Carlos, SP Brazil; 2 Center for Molecular Biology and Genetic Engineering (CBMEG), University of Campinas (UNICAMP), Campinas, SP Brazil; 3 Embrapa Gado de Corte, Campo Grande, MS Brazil; 4 Department of Plant Biology, Biology Institute, UNICAMP, Campinas, SP Brazil; 5 Present Address: Department of Biochemistry, Center of Biological Sciences, Federal University of Santa Catarina, Florianopolis, SC Brazil

**Keywords:** *Brachiaria*, *de novo* transcriptome assembly, molecular markers, RNA-seq, tropical grasses

## Abstract

**Background:**

*Urochloa humidicola* (Koronivia grass) is a polyploid (6x to 9x) species that is used as forage in the tropics. Facultative apospory apomixis is present in most of the genotypes of this species, although one individual has been described as sexual. Molecular studies have been restricted to molecular marker approaches for genetic diversity estimations and linkage map construction. The objectives of the present study were to describe and compare the leaf transcriptome of two important genotypes that are highly divergent in terms of their phenotypes and reproduction modes: the sexual BH031 and the aposporous apomictic cultivar BRS Tupi.

**Results:**

We sequenced the leaf transcriptome of Koronivia grass using an Illumina GAIIx system, which produced 13.09 Gb of data that consisted of 163,575,526 paired-end reads between the two libraries. We *de novo-*assembled 76,196 transcripts with an average length of 1,152 bp and filtered 35,093 non-redundant unigenes. A similarity search against the non-redundant National Center of Biotechnology Information (NCBI) protein database returned 65 % hits. We annotated 24,133 unigenes in the Phytozome database and 14,082 unigenes in the UniProtKB/Swiss-Prot database, assigned 108,334 gene ontology terms to 17,255 unigenes and identified 5,324 unigenes in 327 known metabolic pathways. Comparisons with other grasses via a reciprocal BLAST search revealed a larger number of orthologous genes for the *Panicum* species. The unigenes were involved in C4 photosynthesis, lignocellulose biosynthesis and flooding stress responses. A search for functional molecular markers revealed 4,489 microsatellites and 560,298 single nucleotide polymorphisms (SNPs). A quantitative real-time PCR analysis validated the RNA-seq expression analysis and allowed for the identification of transcriptomic differences between the two evaluated genotypes. Moreover, 192 unannotated sequences were classified as containing complete open reading frames, suggesting that the new, potentially exclusive genes should be further investigated.

**Conclusion:**

The present study represents the first whole-transcriptome sequencing of *U. humidicola* leaves, providing an important public information source of transcripts and functional molecular markers. The qPCR analysis indicated that the expression of certain transcripts confirmed the differential expression observed *in silico*, which demonstrated that RNA-seq is useful for identifying differentially expressed and unique genes. These results corroborate the findings from previous studies and suggest a hybrid origin for BH031.

**Electronic supplementary material:**

The online version of this article (doi:10.1186/s12864-016-3270-5) contains supplementary material, which is available to authorized users.

## Background

Tropical pastures comprise plants from several genera of grasses and legumes [1]. Brazil has the second largest effective cattle herd and the highest beef exports worldwide [2], with 190 million ha of pastures [3]. However, most of the areas cultivated with forage crops are established with a limited number of exotic and clonal reproduction cultivars [4], representing a high risk of genetic vulnerability for forage production.

To minimize the risk of planting large contiguous areas and diversify Brazilian pastures, genetically improved forage species and new cultivars must be established [5]. The *Urochloa* (Poaceae) genus is primarily native to tropical African savannas and widely used for pastures in the tropics. This genus comprises approximately 120 identified species [6], with the species *U. brizantha*, *U. decumbens*, *U. ruziziensis* and *U. humidicola* accounting for 85 % of the cultivated pastures in Brazil [1].

*Urochloa humidicola* (Rendle) Morrone & Zuloaga (syn. *Brachiaria humidicola* (Rendle) Schweick) [7] is widely used in the pastures of Brazil and elsewhere in the tropics, particularly in acidic and poorly drained areas [8]. Although the interest of producers in this species has increased, few cultivars, such as Tully, Llanero and BRS Tupi, are available in Brazil. The development and adoption of new Koronivia grass cultivars with a broad genetic base is crucial for the diversification of forage pastures in the tropics and the enhancement of forage production. Thus, hybrids are being selected for release in this country [9, 10], and they are all derived from the sexual genotype BH031 and the facultative apomictic cultivar BRS Tupi, which are two highly divergent genotypes of Koronivia grass [11, 12].

Koronivia grass is an outcrossed and wind-pollinated perennial tropical grass that primarily reproduces through facultative apomixis, although a single sexual genotype has been identified [12, 13]. This species shows variable levels of ploidy (6x to 9x) and a basic chromosome number of x = 6 [14, 15]. This species presents a relatively large and complex genome (1953 Mbp) [16], although limited data are available on the function and structure of this genetic material.

The genetic and genomic influences on the agronomic traits of interest and the mechanisms involved in the genotype-phenotype relationships in this species are poorly understood [17]. A search for *Urochloa* in the National Center of Biotechnology Information (NCBI) database revealed 2,237 expressed sequence tags (ESTs) as of March 2015. The generation of a reference transcriptome is a critical step for exploring the molecular machinery of a species with few genomic resources, such as *U. humidicola*. RNA-seq is an efficient method of analyzing transcriptomes, generating comprehensive and in-depth biological resources [18, 19] and providing new insights into gene expression patterns. High-throughput sequencing methods produce millions of short sequence reads that facilitate the profiling of gene expression, the discovery of novel transcribed regions, the detection of alternative splicing isoforms, and the identification of valuable molecular markers, such as microsatellites and single nucleotide polymorphisms (SNPs). Therefore, these methods have the potential for use in molecular breeding strategies to improve the productivity and quality of forage [20].

In the present study, we describe the initial sequencing and *de novo* assembly of the transcriptome of *U. humidicola* leaves from two highly divergent genotypes (the sexual accession BH031 and the facultative apomictic cultivar BRS Tupi) that are important for the species biology and breeding using an Illumina massive parallel sequencing platform. The objectives of the present study were to provide novel genetic resources for the species through the *de novo* assembly of RNA-seq data from the leaves of two divergent genotypes, assess the transcriptomic differences between the genotypes and identify potential functional molecular markers because the genomic single sequence repeat (SSR) developed for *U. humidicola* [11, 21–23] displays amplification issues in the sexual genotype BH031.

## Methods

### Plant materials and DNA and RNA extraction

**Fig. 1:**
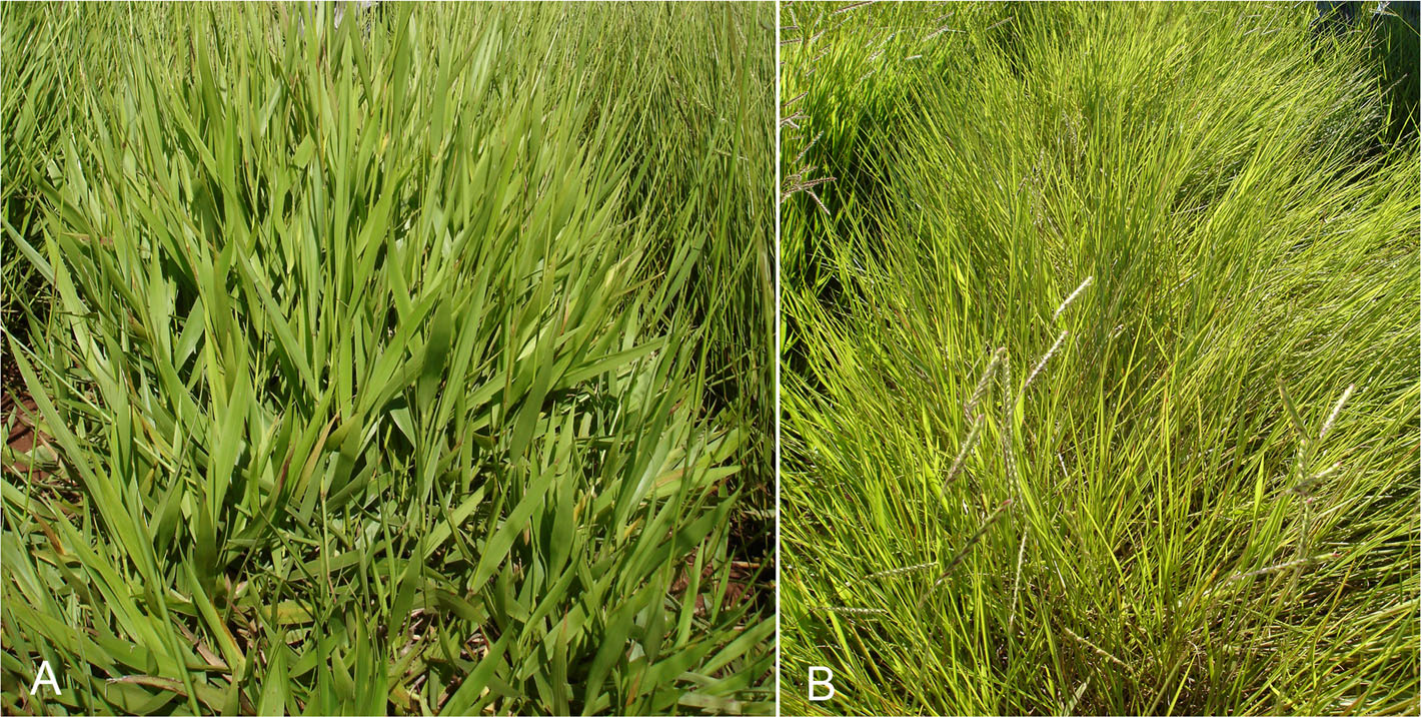
*Urochloa humidicola* genotypes sequenced. **a**) Sexual accession BH031 and **b**) apomictic cv. BRS Tupi; both are maintained at the germplasm bank at Embrapa Gado de Corte

Two divergent genotypes of *U. humidicola* were sampled for RNA sequencing: the sexual accession BH031 and the facultative apomictic cultivar BRS Tupi. BRS Tupi stands out as an option for low-fertility pasture soils subjected to temporary flooding [24]. In addition to their modes of reproduction, both genotypes differ in terms of growth habit, tillering intensity, leaf width and productivity as shown in Fig. 1. These genotypes are the genitors of a mapping population at Embrapa Beef Cattle in Campo Grande, MS, where the genotypes were sampled under previously described conditions [10] and at the same time of day.

On May 12, 2012 (rainy season in Brazil), young leaves were collected from each genotype and immediately placed in RNAlater (Life Technologies, Carlsbad, CA, USA) for stabilization and storage. The samples were transported to the Multiuser Laboratory of Genotyping and Sequencing (LMGS) at the Center of Molecular Biology and Genetic Engineering (CBMEG/UNICAMP, SP, Brazil) and stored at −80 °C until further use. Total RNA was isolated using a modified lithium chloride protocol [25]. We assessed the integrity and quantity of the obtained RNA using a 2100 Bioanalyzer (Agilent Technologies, Palo Alto, CA, USA). Equal molar quantities of high-quality RNA from each material were used as templates for the cDNA synthesis in the subsequent steps.

For the quantitative RT-PCR, young leaves were collected from three different plants of each genotype on January 26, 2015. Total RNA [25] and total DNA [26] were extracted as previously described. The purity and concentration of the RNA were determined using a NanoVue Plus spectrophotometer (GE Healthcare, Piscataway, NJ, USA), and the purity and concentration of the DNA were determined using a NanoDrop 2000/2000c spectrophotometer (Thermo Fisher Scientific, Waltham, MA, USA).

### Preparation and sequencing (RNA-seq) of the cDNA library

A cDNA library was constructed separately for each sample using a TruSeq RNA Sample Preparation Kit (Illumina Inc., San Diego, CA, USA) according to the manufacturer’s instructions. The libraries´ quality was confirmed using a 2100 Bioanalyzer (Agilent Technologies, Palo Alto, CA, USA) and quantified via qPCR (Illumina protocol SY-930-10-10). Clustering was conducted using a TruSeq PE Cluster Kit on cBot (Illumina Inc., San Diego, CA, USA). Subsequently, the cDNA libraries were sequenced, with each one in a single lane, using an Illumina Genome Analyzer IIx with TruSeq SBS 36-Cycle kits (Illumina, San Diego, CA, USA) according to the manufacturer’s specifications for 80-bp PE reads.

### Data filtering and *de novo* assembly

The raw data that were generated from the Illumina sequencer were initially obtained in BCL format and subsequently converted to qSeq format using the Off-Line Basecaller v.1.9.4 (OLB) software. We further converted the qSeq files into FastQ files containing 80-bp reads using a custom script. Filtering for high-quality (HQ) reads was performed using the NGS QC Toolkit 2.3 [27]. Initially, low-quality reads (Phred quality score < 20 in 75 % of all reads) and reads with fewer than 60 bases were removed. Subsequently, the remaining reads were trimmed at the 3’ end, and bases with Phred scores < 20 were removed. All reads were deposited in the NCBI Short Read Archive (SRA) under accession number SRP065020, at the following link: https://www.ncbi.nlm.nih.gov/sra/?term=SRP065020.

In the present study, we compared the performance of two commonly used *de novo* assemblers. Only HQ paired-end (PE) reads from the two sequenced samples were used for assembly and combined together to obtain a representative dataset for the *U. humidicola* leaf transcriptome. We used Trinity (Trinity-r2012-10-05 version) [28] and CLC Genomics Workbench (v4.9; CLC Bio, Cambridge, MA, USA) software for the *de novo* transcriptome assembly from short reads using de Bruijn graphs, for which the reads were initially broken into smaller fragments referred to as k-mers (k denotes the length of these sequences) [28, 29] and subsequently assembled into larger sequences without using a reference genome or transcriptome. Only the default k-mer length (25-mer) was selected for the Trinity assembler, whereas two different k-mer lengths (25-mer and 45-mer) were selected for the CLC Genomics Workbench assembler. The default settings were used for both software programs. To assess the integrity of the assembled transcriptomes, HQ short reads were mapped back into the transcript dataset using the Bowtie sequence aligner [30] with default parameters. We considered various parameters, including the total number of contigs (>300 bp), percentage of mapped reads, length of N50 contigs and average length of the contigs, to select the optimal assembly.

### Functional annotation

After a comparison between the assemblies and selection of the Trinity assembly, which was the optimal method, we further selected distinct sequences (unigenes) among the complete dataset of transcripts. The Trinity assembly has three modules that are executed in sequence (Inchworm, Chrysalis, and Butterfly), and the unigenes were filtered using criteria that retain the first Butterfly transcript generated per Chrysalis component, which is considered representative.

The unigenes were compared against the NCBI non-redundant protein (Nr) database and the UniProtKB/Swiss-Prot database [31]. A homology search against these databanks was performed using the BLASTX option from the BLAST+ suite [32] with an e-value cutoff of 1e-06.

We constructed a grass transcriptome databank that contained several species (*Brachypodium distachyon, Oryza sativa, Panicum hallii, Panicum virgatum, Sorghum bicolor,* and *Setaria italica*) using Phytozome v9.0 [33] and the previously developed *Panicum maximum* transcriptome [34]. The unigenes were compared with this grass databank using BLASTN (e-value cutoff of 1e-10) from the BLAST+ suite and a custom script to generate reciprocal BLAST hits (RBH), and the putative orthologous relationships were determined. This approach was also used to individually compare each species.

Gene ontology (GO) [35] terms were retrieved from the sequence that was functionally annotated in the NCBI nr databank using Blast2GO software [36]. The GO terms were mapped to each annotated transcript based on the 10 best hits, after which we proceeded to the annotation step in the Blast2GO software using default parameters with the exception of the e-value cutoff (1e-10). The GO annotation was enriched using ANNEX [36], and we used Go-slim [35] with plant slim (*Arabidopsis thaliana*) as an alias to summarize the GO terms of the transcriptome and facilitate data interpretation. WEGO [37] was used for the graphical representation of the GO functional classification, demonstrating the distribution of *U. humidicola* gene functions into the three main ontology categories: biological processes, molecular functions, and cellular components. An enrichment analysis using Fisher’s exact test (FDR < 0.05) in the Blast2GO software was applied to search for enriched GO terms between the unique transcripts of BH031 and BRS Tupi and between the unique transcripts of *U. humidicola* (compared with the grass databank) and the assembled transcriptome in the present study. Unigenes were assigned to known metabolic pathways using the Kyoto Encyclopedia of Genes and Genomes (KEGG) database [38]. The KEGG Automatic Annotation Server (KAAS) [39] was used to obtain assignments with the bidirectional best hit (BBH) method, which generated KEGG Orthology (KO) terms related to the assembled leaf transcriptome. The sequences were matched against the Pfam database [40] using the InterProScan tool [41] to identify the protein domains of the assembled unigenes. Moreover, we searched the assembled dataset of transcripts for open reading frames (ORFs) using TransDecoder (http://transdecoder.github.io/) with a minimum length of 100 amino acids (aa) and default parameters.

### Read mapping and transcript abundance analyses

We used RNA-seq by Expectation Maximization (RSEM) [42] to estimate the transcript expression levels. The analyses were performed using combined data from the samples and the data from each genotype separately based on the number of fragments that were mapped to the Trinity assembly contigs. The transcript FPKM values (fragments per kilobase of transcript per million mapped reads) were estimated using RSEM with Bowtie read mapping [30]. We used the RSEM analysis as the main criteria to differentiate the unigene distribution along the two sequenced genotypes, and only transcripts with FPKM values larger than 0.5 were considered to identify unique and shared transcripts. A Venn diagram was obtained using Venny 2.0.2 [43].

### Putative molecular markers

The MISA (MIcroSAtellite) script [44] was used to identify SSRs. Microsatellite regions were defined as containing at least six motif repetitions for dinucleotides and four motif repetitions for each tri/tetra/penta/hexa-nucleotide motif. A SSR motif was defined as a compound when two or more SSR motifs were interrupted by up to 50 bp.

To identify putative SNP positions, the CLC Genomics Workbench was used to map reads to the *de novo* assembled leaf transcriptome. The parameters were as follows: length fraction = 0.9, similarity = 0.9, mismatch cost = 2, insertion cost = 3, and deletion cost = 3. Subsequently, the putative SNPs were detected using the following criteria: window length = 11, minimum coverage = 20, minimum central base quality score = 30, minimum average quality score = 20, minimum coverage at SNP site = 20, minimum frequency = 10 %, and ploidy = 6. Default settings were used for the remaining parameters.

### Quantitative RT-PCR analysis

To validate the RNA-seq expression analysis, transcripts that displayed different expression patterns *in silico* were selected for quantitative RT-PCR analysis based on the RSEM quantification. Primer3Plus software [45] was used to design primer pairs for the sequences. The target amplicon size was set at 70–150 bp, and the optimal annealing temperature was 60 °C and optimal primer length was 20 bp. Reference genes were selected according to previous studies in grasses [46–49], searched in the *U. humidicola* transcriptome, and used to design the primers as described above. All of the primer pairs were initially evaluated by PCR using genomic DNA as a template. The primer pairs that successfully amplified the DNA of both genotypes with an amplification efficiency of 90-110 % by qPCR were used for the expression analysis.

Three biological replicates for each of the two genotypes were used for the qPCR assays. Total RNA (500 ng) was used for cDNA synthesis using the QuantiTect Reverse Transcription Kit (Qiagen Inc., Chatsworth, CA, USA), which includes a genomic DNA removal step. The cDNAs were diluted (1:20) in nuclease-free water, and 2 μL samples were used for the qPCR reactions.

Quantitative RT-PCR was performed using a CFX384 Real-Time PCR Detection System with iTaq Universal SYBR® Green Supermix (Bio-Rad Laboratories Inc., Hercules, CA, USA) according to the manufacturer’s instructions, and the final primer concentration was 0.3 μM. The reaction conditions were 95 °C for 10 min and then 40 cycles of 95 °C for 30 s and 60 °C for 1 min. No template controls for either primer pair were included, and each reaction was performed in triplicate. The BRS Tupi genotype sample was used as the control for the normalization of gene expression. The presence of single amplicons in the PCR products was confirmed by a melting curve analysis with temperatures ranging from 65 °C to 95 °C at increments of 0.5 °C. The baseline and Cq values were automatically determined, and the expression analysis (ΔΔCt method) was performed using CFX Manager 2.1 software (Bio-Rad Laboratories, Inc., USA).

Reference genes were selected according to the amplification efficiency (E = 90-110 % and R^2 > 0.99), gene expression stability values (M < 0.5) and coefficients of variance (CV < 0.25) among the samples. Statistical significance was tested using a pair-wise fixed reallocation randomization test (10,000 iterations) with the relative expression software tool (REST 2009, Qiagen) [50]. Differences were considered statistically significant at *p* < 0.05.

## Results and discussion

### Sequencing and filtering

In the present study, we performed a *de novo* assembly of the *U. humidicola* leaf transcriptome and characterized the transcriptome at the single nucleotide level. Each genotype was sequenced independently using the Illumina GAIIx platform (Table 1), which resulted in a total of 163,575,526 (13.09 Gb) PE reads from both libraries combined. Sequencing of the cv. BRS Tupi genotype produced a slightly larger number of reads (54.85 % of total reads) compared with that of the BH031 genotype (45.15 % of total reads). However, this difference did not interfere with the assembly or characterization of the *U. humidicola* leaf transcriptome. The short read filtering process resulted in a total of 133,201,738 (10.66 Gb) high quality (HQ) PE reads from both libraries, and these sequences were used for the *de novo* assembly. The genotype-filtered data are shown in Table 1, and they demonstrate that BRS Tupi retained more HQ reads than did BH031 after the cleaning and filtering procedures; however, this difference was not significant.

**Table 1:** Summary of Illumina sequencing output statistics

		BH031	BRS Tupi	Total
Sequenced	Number of reads	73779964	89795562	163575526
	Total bases (Gb)	5.90	7.18	13.09
Filtered	Number of reads	57322934	75878804	133201738
	Total bases (Gb)	4.59	6.07	10.66
	High quality data	77.8 %	84.5 %	81.4 %

### *De novo* transcriptome assembly

We compared the results of the Trinity and CLC Genomics Workbench assemblers because these methodologies use different approaches for the *de novo* assembly of short reads. For the CLC Genomics Workbench assemblies, we observed that the total number of assembled contigs, the mean length and the N50 length displayed better metrics when using a k-mer value of k = 25 compared with k = 45 (Table 2); however, an increase in the k-mer value to k = 45 led to a slightly greater improvement in the percentage of mapped reads. The Trinity assembly displayed the largest values for the total assembled transcripts, mean length and N50 length (Table 2). The Bowtie aligner, which only considered properly mapped paired ends, mapped 82.94 % of the reads onto assembled sequences. Moreover, the Trinity assembler provided isoform data on the transcripts, which will be valuable for future studies. Thus, the Trinity assembly was preferable to the CLC Genomics workbench assembly.

**Table 2:** Comparison of the *de novo* assemblies generated using the Trinity and CLC Genomics Workbench programs

	Trinity	CLC Genomics Workbench	
K-mer (bp)	25	25	45
Total number of contigs	76196	51291	44899
Min contig length (bp)	301	300	300
Max contig length (bp)	7392	6174	5506
Mean contig length (bp)	877	617	551
Median contig length (bp)	637	454	435
N50 (bp)	1152	665	562
Mapped reads (%)	82.94	79.53	80.23

Considering the assembly metrics that were obtained using the short reads generated using the Illumina platform, the N50 contig length of the *U. humidicola* leaf transcriptome was greater than that of *Panicum maximum* (981 bp) [34] and bamboo (1,132 bp) [51]. In addition, the average length of the unigenes was greater than that of *P. maximum* (758 bp) [34], *Salvia splendens* (772 bp) [52], *Youngia japonica* (795 bp) [53] and *Houttuynia cordata* (679 bp) [54], suggesting that the quality of the assembly from Illumina PE sequencing for this non-model organism was satisfactory compared with that of the other *de novo* assemblies.

A total of 35,093 sequences (46.12 % of all transcripts) was filtered as unigenes from the Trinity assembly using established criteria, which resulted in a dataset with a mean length of 870 bp, a N50 contig length of 1,171 bp and a GC content of 47.08 %. We obtained 7,588 (21.62 %) unigenes that were longer than 1 kb, a size range that commonly confers a high annotation rate [34, 55]. We also compared the unigene metrics obtained here to the transcriptomes that were obtained from Phytozome (Additional file 1) and found that the observed total contig number, N50 contig length, average contig length and GC content more closely resembled the Phytozome transcriptomes of these species. It is important to note that the Phytozome datasets are more curated and constantly updated as part of an ongoing process, whereas the present study is an initial attempt to characterize the *U. humidicola* leaf transcriptome.

### Annotation

**Fig. 2:**
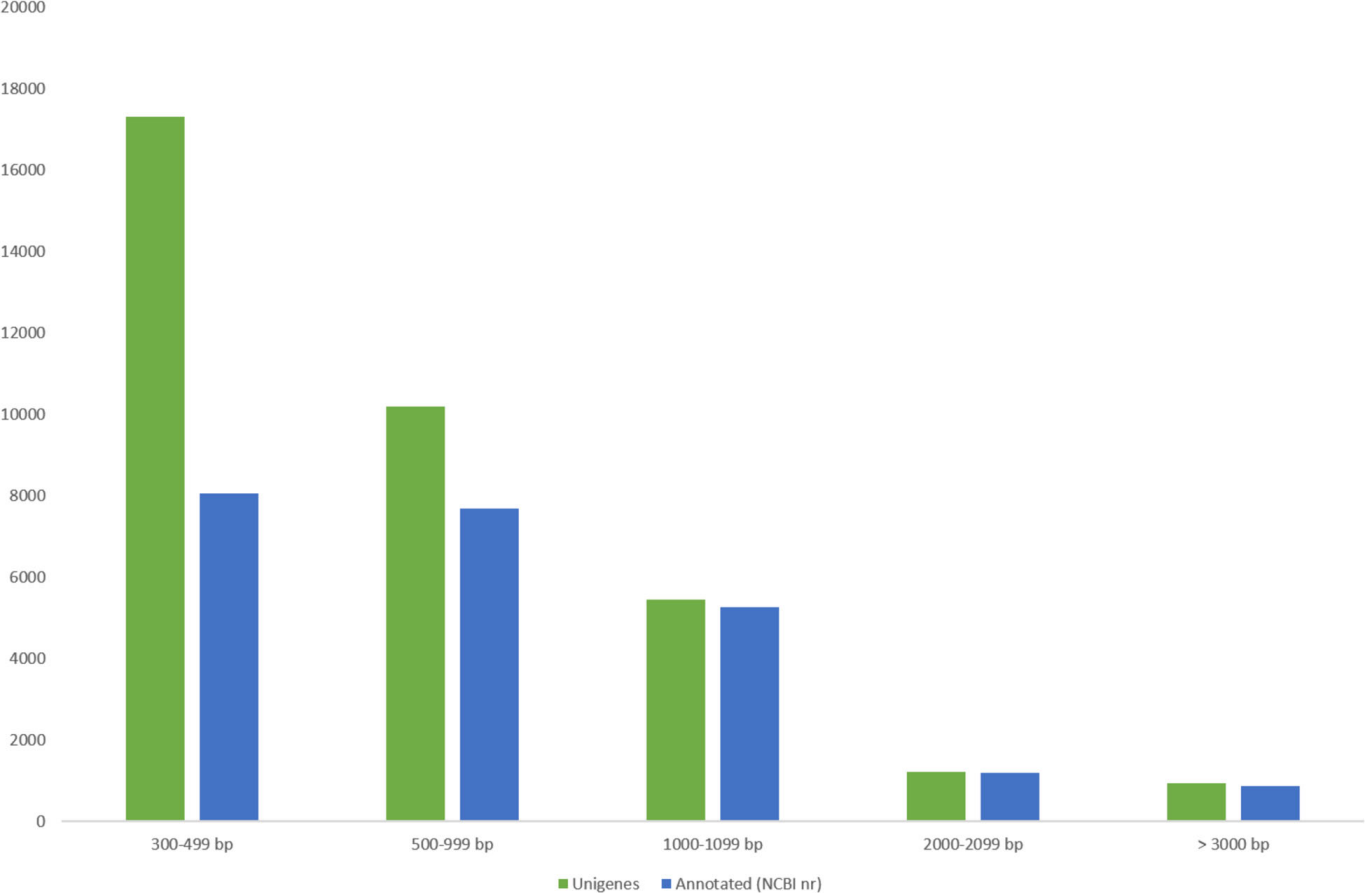
Comparison of the size ranges of the unigenes and annotated (NCBI nr) unigenes

**Fig. 3:**
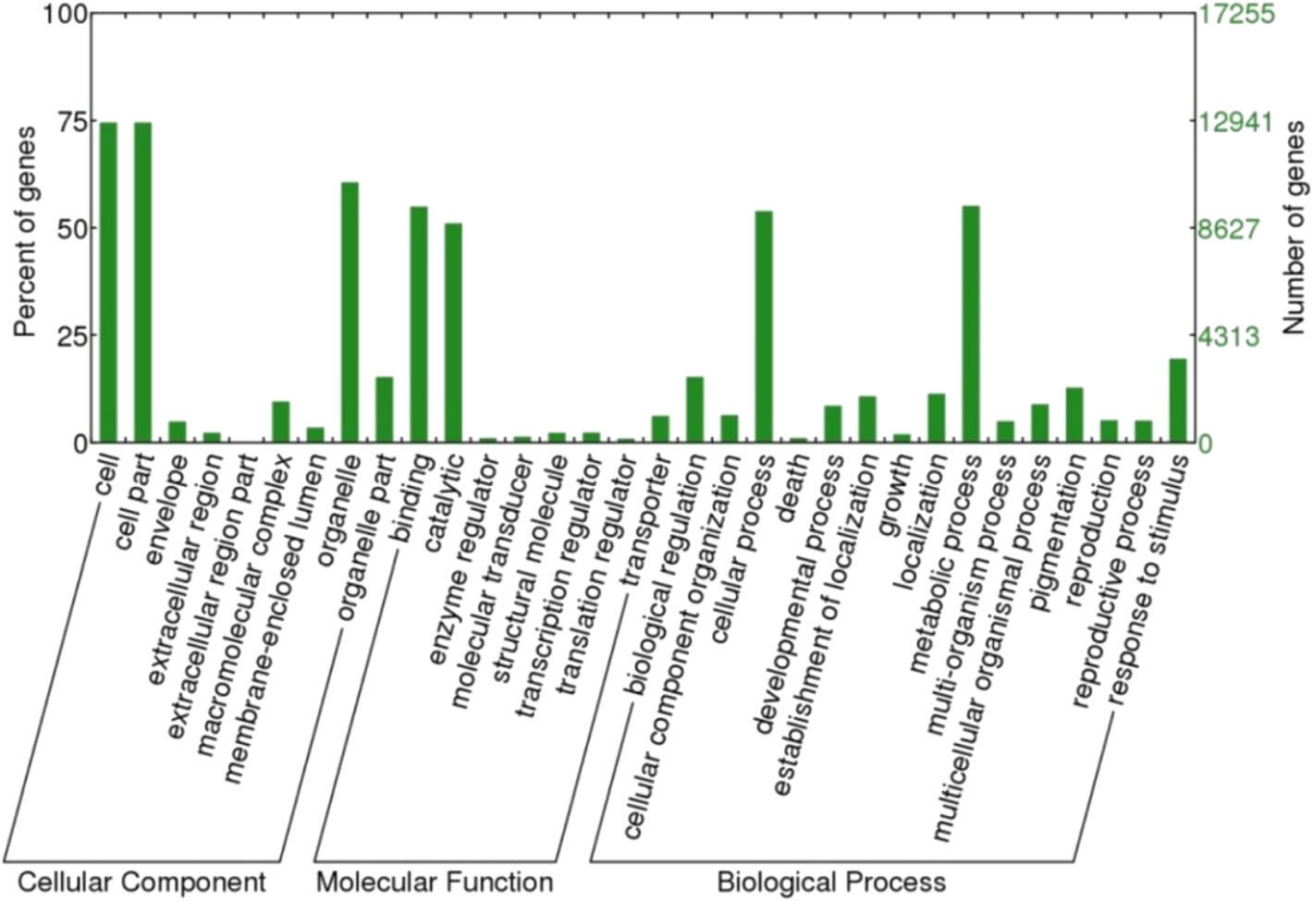
GO classification for the *U. humidicola* leaf transcriptome

We performed a unigene homology search using several databases, including the NCBI non-redundant protein database, the UniProtKB/Swiss-Prot database, a custom grass database, and the Kyoto Encyclopedia of Genes and Genomes (KEGG), and we retrieved GO terms and Pfam protein domains. Table 3 shows the overall results of the annotation. Among the 35,093 unigenes, 23,071 (65.7 %) displayed homology to sequences in the NCBI nr database. Most of these unigenes were related to proteins from *Sorghum bicolor* (9,866), followed by proteins from *Zea mays* (7,134), *Oryza sativa* (2,631), *Brachypodium distachyon* (701) and *Aegilops tauschii* (563) (Additional file 2). These five species accounted for ~90 % of the total top hits in the NCBI nr databank comparison, and this result was expected because these species are closely related to *U. humidicola* and classified within Poaceae, which includes species with a high level of genomic synteny [56]. As expected, unigenes with a size > 1 kb showed a higher annotation rate (96.6 %) compared with unigenes with a size less than 1 kb (57.2 %) (Fig. 2). Based on the NCBI nr annotation, 17,255 (49.2 %) unigenes had assigned GO terms, and these terms were divided into three main classes of ontologies: cellular component, molecular function and biological process. The majority of the GO terms were assigned to the terms cellular component (45,259 terms, 41.78 %) and biological process (41,785 terms, 38.57 %), whereas a smaller number were assigned to the term molecular function (19.64 %). Regarding the cellular component class, 13 different terms were obtained, and proteins located in the cell and cell parts were dominant and presented same number of unigenes (13,135) because these GO terms are typically assigned together. In the molecular function class, proteins related to binding (9,483) and catalytic activity (8,805) were primarily observed for the *U. humidicola* unigenes, and these classes included 14 different terms. Moreover, the biological process ontology primarily consisted of proteins involved in cellular and physiological processes, and the terms cellular process (9,722 unigenes) and metabolic process (9,707 unigenes) were the most representative GO terms followed by response to stimulus (3,470 unigenes), for which the response to stress subterm (2,216 unigenes) was the most representative category (Fig. 3).

**Table 3:** Summary of the annotated *U. humidicola* transcriptome

	No. of annotated unigenes	% of annotated unigenes
NCBI nr	23,071	65.7 %
UniProtKB/Swiss-Prot	14,082	40.1 %
Gene Ontology	17,255	49.2 %
KEGG	5,324	15.2 %
Pfam	10,880	31.0 %

**Fig. 4:**
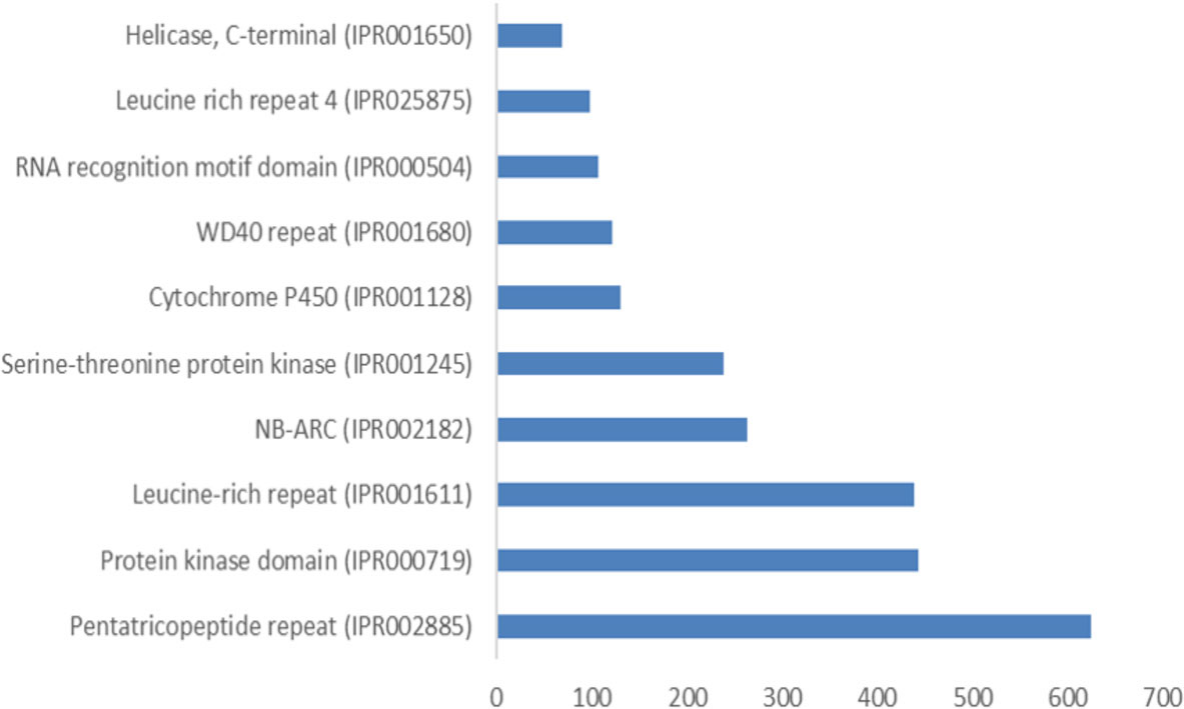
Distribution of the top 10 Pfam domains identified in the *U. humidicola* unigenes

We also identified 3,024 different Pfam protein domains in 10,880 unigenes using InterProScan. The top 10 Pfam domains are presented in Fig. 4. The most abundant protein domain was the pentatricopeptide repeat (PPR), which is characterized by tandem repeats of a degenerate 35-aa-long motif [57]. PPRs play a role in post-transcriptional processes within organelles and possess sequence-specific RNA-binding properties. Plant genomes contain between one and five hundred PPR genes per genome, whereas non-plant genomes encode two to six PPR proteins [58]. We identified 624 unigenes with PPR domains in the present study. An interesting abundant Pfam domain was NB-ARC, a signaling motif that is shared by plant resistance gene products and regulators of cell death in animals [59]. Additionally, several Cytochrome P450 (CYP450) domains were identified among the unigenes. Cytochrome P450 enzymes are a superfamily of mono-oxygenases that are observed in all kingdoms of life, and they play several roles in metabolism and reflect increased biochemical diversity. In plants, CYP450s are involved in the biosynthesis of several compounds, such as hormones, defensive compounds and fatty acids [60].

To correlate *U. humidicola* unigenes with known metabolic pathways, we used the KAAS server to assign sequences with KO terms and their respective KEGG maps. A total of 5,234 (15.2 %) assembled unigenes were associated with 3,936 KO terms and 327 pathways. The primarily represented pathways were metabolic pathways (750 members) and secondary metabolite biosynthesis pathways (313 members). Other important pathways included the glycolysis/gluconeogenesis pathway (Additional file 3) and plant hormone signal transduction pathway (Additional file 4). The KAAS results revealed that the assembled unigenes were distributed among several metabolic pathways.

The transcripts and unigenes were submitted to an ORF predictor using TransDecoder, and we detected ORFs in 53.85 % of the assembled transcripts and in 61.64 % of the unigenes. Further information regarding the ORFs is provided in Additional file 5.

### Transcript abundance analysis

Next-generation sequencing provided an overview of the genomic expression profile in the leaves of *U. humidicola* at a specific moment. The mean FPKM value for all of the unigenes was ~29. We selected the ten most abundant transcripts expressed in the leaves (Table 4), and the results showed that the common expression pattern was associated with photosynthesis, defense mechanisms and stress responses, which is consistent with results obtained for the *P. maximum* transcriptome [34]. The most represented unigene was Photosystem II reaction center protein M (psbM), which had a FPKM value of 12,514.83. This protein is involved in photosynthesis reactions in photosystem II, which uses light energy to abstract electrons from water to generate a proton gradient that is involved in ATP formation [61]. The second-most abundant unigene was associated with an uncharacterized ycf68 protein. This protein is encoded in the chloroplast and has no known function; however, because this family is exclusively observed in the chloroplasts of phototrophic organisms, this protein might play a role in photosynthesis. In addition to these genes, the DNA-binding protein MNB1B was highly represented. This protein recognizes the AAGG motif at the MNF1 binding site. MNF1 is a nuclear factor that interacts with the promoter region of the phosphoenolpyruvate carboxylase (PEPC) gene, which is involved in the catalysis of the primary fixation of CO_2_ during C4 photosynthesis [62]. The remaining unigenes were described as participating in the defense mechanisms and stress responses of the plant, including the gene encoding metallothionein-like protein 1A. Metallothioneins are low-molecular-weight cysteine-rich proteins that coordinate metal atoms and are induced under different stress conditions, such as excess heavy metal exposure, heat shock and salt stresses, where these proteins play important roles in maintaining intracellular metal homeostasis, eliminating metal toxicity and protecting against intracellular oxidative damage [63, 64]. The protein thiamine thiazole synthase 2, chloroplastic (THI42) was also abundant, and it has been implicated in the synthesis of the essential cell nutrient thiamine (vitamin B1), or more precisely, the thiazole ring (thiamine precursor) [65]. Moreover, THI42 may play additional roles in adaptation to various stress conditions and DNA damage tolerance. The protein TIFY 10A, which is also known as jasmonate ZIM domain-containing protein 1 (JAZ1), represses the transcription of jasmonate-responsive genes. Thus, this protein is a repressor of jasmonate (JA), an essential phytohormone in plants. The perception of bioactive JAs through the F-box protein coronatine insensitive1 (COI1) leads to the degradation of JAZ1 via the ubiquitin-proteasome pathway, which in turn activates the expression of genes that are involved in plant growth, development, and defense [66]. Cysteine proteases are commonly present in plants and expressed in various organs. These enzymes are involved in digestion, post-translational modification of storage proteins, antibiotic responses and programmed cell death [67]. Specifically, cysteine proteinase 1 is responsible for the degradation of the storage protein zein and may play a role in proteolysis during emergencies. The last unigene among the ten most abundant identified genes was related to the putative polyamine oxidase 2, a flavoenzyme that catalyzes the oxidation of the secondary amino group of polyamines. Polyamine oxidase 2 is located in peroxisomes, which are organelles that are involved in various stress responses [68]. Furthermore, the putative uncharacterized protein ART2 (ribosomal RNA transcript antisense to protein 2) was observed, and among the ten unigenes with higher FPKMs, this transcript did not show similarity to any protein in the Swiss-Prot database. Examination of this unigene for the presence of candidate coding regions revealed a complete ORF. Using this unigene to search against the Phytozome grass data revealed similarity (e-value of 4e-18) with an uncharacterized protein (hypothetical gene #37831442) from *Setaria italica*, *Oryza sativa* and *Brachypodium distachyon*. These unigenes represent genes that have not yet been described, and additional accurate molecular and proteomic analyses are required to validate and determine the functions of these genes.

**Table 4:** Ten most abundant transcripts identified in the *U. humidicola* leaf transcriptome

Putative gene	E-value	FPKM	UniProtKB
photosystem II reaction center protein M	7.00E-14	12514.83	sp|A1E9R2|PSBM_SORBI
uncharacterized ycf68 protein	--	3330.18	sp|P12173 |YCF68_ORYSJ
DNA-binding protein MNB1B	8.00E-52	3004.90	sp|P27347|MNB1B_MAIZE
putative uncharacterized protein ART2	3.00E-16	2583.30	sp|Q8TGM7|ART2_YEAST
metallothionein-like protein 1A	4.00E-19	2575.12	sp|P0C5B3|MT1A_ORYSJ
thiamine thiazole synthase 2, chloroplastic	0.0	2422.49	sp|C5X2M4|THI42_SORBI
protein TIFY 10A	2.00E-25	2167.52	sp|Q9LMA8|TI10A_ARATH
cysteine proteinase 1	0.0	2144.41	sp|Q10716|CYSP1_MAIZE
No-hit	--	2090.78	--
probable polyamine oxidase 2	0.0	2076.58	sp|Q9SKX5|PAO2_ARATH

### Comparison with grass transcriptomes

We searched for orthologs of the *U. humidicola* unigenes by comparing the unigenes of several grass species (species used in the custom databank in the annotation step) to identify unique transcripts for this forage plant. Reciprocal BLAST hits returned 24,133 transcripts with putative orthologous relationships. Compared with each individual grass species in the databank, *U. humidicola* unigenes returned more hits from the *P. maximum* transcriptome (15,427 hits or 43.96 % of the unigenes), whereas *Brachypodium distachyon* returned fewer hits (8,768 hits or 24.98 % of the unigenes) (Additional file 6).

**Fig. 5:**
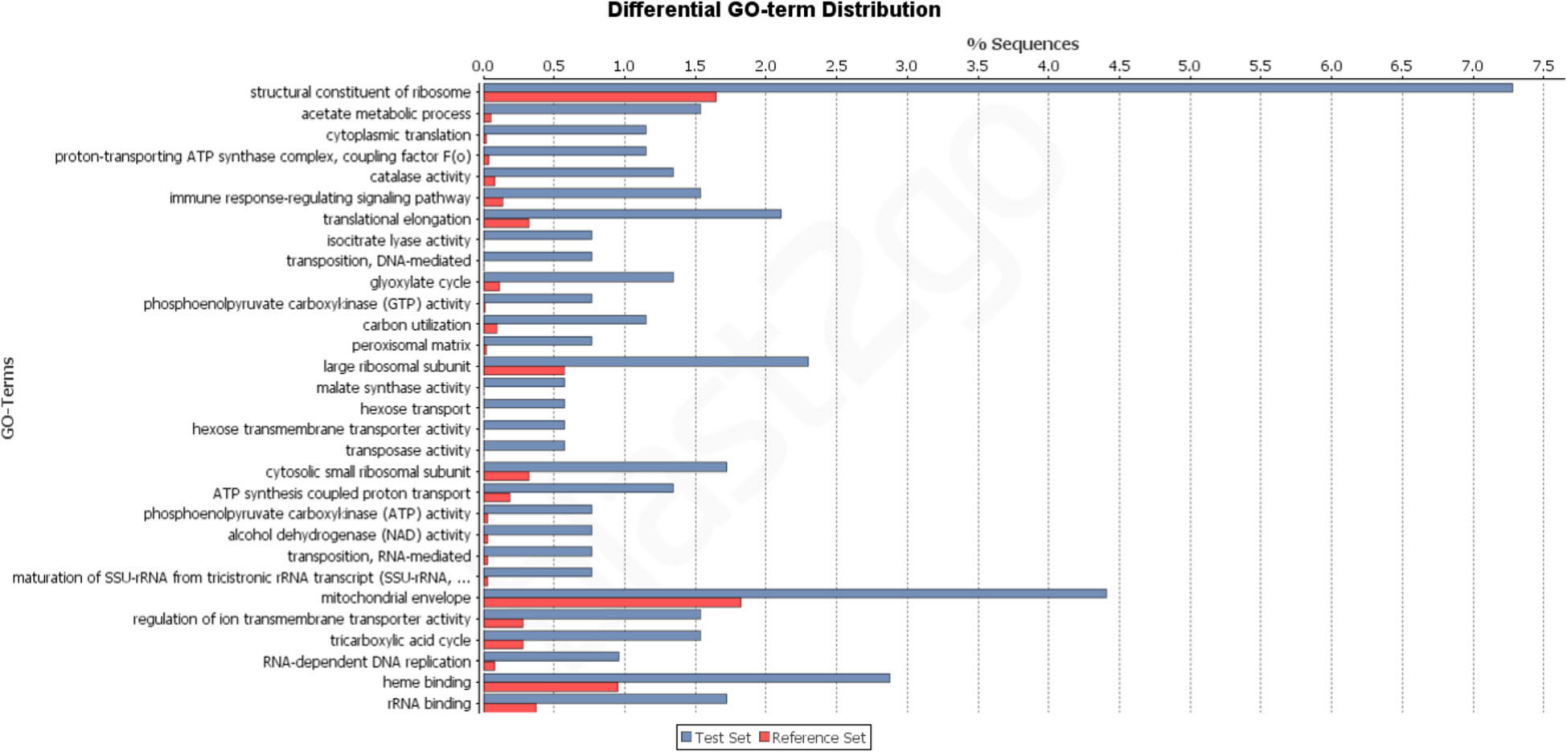
Enriched gene ontology (GO) terms for the unique *U. humidicola* unigenes

Regarding the unigenes without a relationship to the transcriptomes of other grasses (10,960 unigenes or 31.23 %), 2,231 unigenes were annotated in NCBI nr and 1,010 unigenes were annotated in UniProtKB/Swiss-Prot. We selected these 10,960 unigenes to identify enriched GO terms relative to that of all *U. humidicola* unigenes (Fig. 5). Several classes associated with oxidative stress were identified, including carbon utilization, immune response signaling and catalase activity. Among the 8,729 unigenes that were unique to *U. humidicola* but not annotated, 980 unigenes had ORFs, among which 192 unigene sequences were complete ORFs. This result warrants further investigation of new genes that are potentially exclusive to Koronivia grass.

### Genes involved in flooding stress

Most crops and wild plants cannot tolerate floods and suffer from severe growth reduction or even death under these conditions [69]. However, *U. humidicola* is recognized for its tolerance to poorly drained soils and seasonal flooding as well as infertile acidic soils [8]. Although the leaf transcriptome analysis in the present study was not performed under flood stress, we identified genes that are involved in the flooding stress response (Additional file 7), including genes that code for plant hormones, particularly ethylene, ethylene precursors (synthesized through 1-aminocyclopropane-1-carboxylic acid (ACC) synthase [70, 71]), and ethylene response sensors [72]. Here, we identified nine unigene representatives of ACC synthase and four representatives of ethylene response sensors (ERS/ETR1). The ethylene signaling pathway is activated in response to low oxygen stress and initiates and regulates many adaptive molecular, chemical and morphological responses. These responses facilitate the avoidance of anaerobiosis by increasing the availability of oxygen to roots in flooded or waterlogged soil, which may occur through the development of aerenchyma [73, 74]. Eight unigenes were associated with ethylene-insensitive protein 2 (EIN2), a central factor in signaling pathways that is regulated by ethylene and necessary for ethylene-mediated gene regulation. We also identified two ethylene insensitive 3-like 1 proteins that are likely putative transcription factors that function as positive regulators of the ethylene response pathway. In addition, 15 unigenes were related to the ethylene-responsive transcription factor, which is completely dependent on a functional EIN2. We also identified unigenes that were associated with alcohol dehydrogenase (ADH), pyruvate decarboxylase (PDC) [75] and lactate dehydrogenase (LDH) [76] genes, which are expressed in response to hypoxia.

### Genes involved in the C4 photosynthesis pathway

Certain grasses, such as *U. humidicola*, exhibit C4-type photosynthesis, which confers elevated plant productivity, particularly at higher temperatures, in addition to higher water and nitrogen use efficiencies [77]. Analysis of the unigenes revealed 11 different genes that were involved in the C4 pathway (Additional file 8), including phosphoenolpyruvate carboxylase (PEPC) (FPKM value 194.84) and carbonic anhydrase (CA) (FPKM value 398.62), which are enzymes responsible for the initial fixation of CO_2_ in the C4 mesophyll cell cytosol and subsequent formation of C4 acid oxaloacetate [78]. The C4 acid oxaloacetate is reduced to either malate or aspartate through the enzymes NADP-malate dehydrogenase and aspartate aminotransferase, respectively, which were also identified in the present study. Subsequently, the decarboxylation reaction occurred, which released CO_2_ in the vicinity of Rubisco, a key step in photosynthetic CO_2_ assimilation in some C4 plants. The results of the present study demonstrated that the phosphorylation of PEPCK (FPKM 1,116) predominantly occurred in *U. humidicola* in relation to NAD-ME (FPKM 280.01) and NADP-ME (FPKM 7.36). Species such as *Urochloa panicoides* and *Panicum maximum* are considered PEPCK-type C4 plants [34, 77]. PPDK was also representative among the unigenes and had a FPKM value of 1,045.

### Genes involved in cellulose and lignin biosynthesis

Cellulose and lignin are the most abundant polymers in nature. Along with hemicellulose, these polymers are the major components of the largest source of plant biomass: the lignocellulose wall [79].

Cellulose (b-1,4-glucan) is the predominant polymer of the lignocellulose wall and provides strength and flexibility to plant tissues. This polymer is of great importance to the wood, paper, textile, and chemical industries [80]. Lignin is a complex and heterogeneous mixture of polymers that is primarily derived from three hydroxycinnamyl alcohols (or monolignols): coniferyl alcohol (guaiacyl propanol “G”), sinapyl alcohol (syringyl propanol “S”) and p-coumaryl alcohol (p-hydroxyphenylpropanol “H”). Lignin is the key element that limits cell wall digestibility in ruminants that consume high-forage diets [81]. In general, dicotyledonous angiosperm (hardwood) lignins principally consist of G and S units and traces of H units, whereas lignins from grasses (monocots) incorporate G and S units at comparable levels and present more H units than dicots [82]. The proportion of G:S:H units in the cell wall is an important characteristic for plant breeders and molecular biologists and a determinant of the successful improvement of forages for livestock feeding.

Cellulose is produced from sitosterol-b-glucoside via certain gene families, such as cellulose synthases (CesA) and glycosyl transferases. In the present study, we identified 18 representatives of cellulose synthase A among the *U. humidicola* leaf unigenes (Additional file 9), and our approach also resulted in the identification of other proteins, including sucrose synthase (SuSy) and UTP--glucose-1-phosphate uridylyltransferase, both of which are associated with five unigenes. The most abundant identified transcript was UDP-glycosyltransferase, which had 53 transcript representatives. In addition to other genes in this pathway, seven genes were related to alkaline/neutral invertase. Invertases have been proposed as substitutes for SuSy in nonphotosynthetic cells [83]. Several other proteins have been implemented in cellulose production, such as COBRA-like protein and chitinase-like protein 1, for which we identified eleven and one related unigenes, respectively.

We also mined the current transcriptomic database to obtain unigenes associated with lignin biosynthesis (Additional file 9). We identified enzymes that were putatively involved in phenylpropanoid metabolism and, consequently, monolignol formation. In addition to these enzymes, 12 sequences were associated with chitinases, five sequences were associated with laccases, 37 sequences were associated with peroxidases and nine sequences were associated with dirigent proteins, which are also important for lignification.

### SSR discovery

Next-generation sequencing technology provides access to a wealth of sequence information. Despite the increasing demand for SNP markers for genotyping, SSR markers continue to be important because of they contain large amounts of genetic information that is primarily relevant to parentage composition or forensic studies, where polyallelic variation is useful [84]. SSR markers play an important role in germplasm characterization, linkage and QTL map construction, gene flow and mating system evaluations, and marker-assisted selection [85, 86]. In *U. humidicola*, the currently available SSR markers [11, 21–23] were all developed from enriched genomic libraries.

The unigenes from *U. humidicola* that were obtained in this study represent a valuable resource for SSR mining. Among the 35,093 unigenes, a total of 4,489 putative SSRs were identified in 3,491 unigenes, 566 of which contained more than one SSR and 191 of which were present in compound form (Table 5). The compilation of all SSRs revealed that one SSR on average was identified for every 6.81 kb of the unigenes.

**Table 5:** Summary of the SSR search results

Search Item	Numbers
Total number of sequences examined	35,093
Total size of examined sequences (bp)	30,553,233
Total number of identified SSRs	4,489
Number of SSR-containing sequences	3,491
Number of sequences containing more than 1 SSR	566
Number of SSRs present in compound formation	191
Dinucleotide	363
Trinucleotide	3,868
Tetranucleotide	173
Pentanucleotide	54
Hexanucleotide	31

**Fig. 6:**
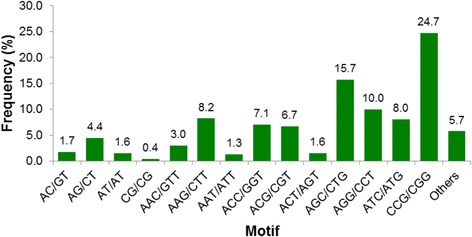
Frequency distribution of SSRs based on the motif sequence types

Regarding the unique unigenes containing SSRs of the two sampled genotypes, 516 exclusive unigenes of BRS Tupi included identified SSRs, whereas the BH031 genotype had 190 exclusive unigenes with detected SSRs. The frequency, type and distribution of the 4,489 potential SSRs were also analyzed in the present study. Among the 4,489 SSRs, trinucleotide repeat motifs were the most abundant (3,868, 86.2 %) (Table 5), which is similar to the *P. maximum* transcriptome (86 %) [34] and the *Brachiaria ruziziensis* genome (85.2 %) [87], followed by dinucleotide (363, 8.09 %), tetranucleotide (173, 3.85 %), pentanucleotide (54, 1.20 %) and hexanucleotide (31, 0.69 %) repeat motifs. The total distribution of SSR motifs was similar to that determined for *P. maximum* [34], whereas in *B. ruziziensis* [87], tetranucleotide motifs were more abundant than dinucleotide motifs (Additional file 10). The dinucleotide to hexanucleotide motifs were further analyzed for SSR repeats. Within the SSRs, 231 motif sequence types were identified, of which the di-, tri-, tetra-, penta- and hexa-nucleotide motifs had 12, 60, 94, 44 and 21 repeats, respectively (Table 6). The most abundant motif detected in the SSRs was the CCG/GGC trinucleotide repeat (1,108, 24.68 %), which is consistent with the results for other grasses, including *P. maximum* [34], *Brachiaria ruziziensis* [87]*, Panicum virgatum* [88, 89] and *Hordeum vulgare* [44]. The remaining 258 types of motifs accounted for 5.75 % of the total SSRs (Fig. 6). In summary, we identified a large number of potential functional SSR markers for which primer pairs can be designed and tested to validate the microsatellite loci.

**Table 6:** Length distribution of the SSRs based on the number of repeats

N. of repeats	Di-	Tri-	Tetra-	Penta-	Hexa-	Total
4	-	2911	135	44	30	3120
5	-	718	34	10	0	762
6	159	168	2	0	0	329
7	92	62	2	0	0	156
8	34	8	0	0	0	42
9	26	1	0	0	0	27
10	20	0	0	0	1	21
11	24	0	0	0	0	24
≥12	8	0	0	0	0	8

### SNP discovery

A total of 560,298 putative SNP positions were identified in 27,739 different unigenes (79.04 % of the total unigenes), which yielded a density of one SNP position per 119 bp (Table 7). This density was slightly lower than that identified in a previous study for *P. maximum*, which was reported to have a density of 1 SNP per ~90 bp; however, in that study, four genotypes were utilized, thereby increasing the total density of the SNP positions [34]. Among the 27,739 unigenes, 1,427 unigenes were unique to sexual accession BH031 and 4,388 unigenes were unique to cv. BRS Tupi (apomictic cultivar). As expected, transition SNPs (Ts) were more common compared with transversion (Tv) SNPs, resulting in a Ts/Tv ratio of 1.86 (Table 7). Among the transversion variations, C ↔ G was the most highly represented, with 52,161 SNPs, and A ↔ T was the least common, with 45,773 SNPs. The direction and strength of selection maintains transitions over transversions among spontaneous mutations because these parameters generate synonymous mutations in coding sequences [90]. We also identified SNPs in the sequences of unigenes involved in the key pathways described herein. The 59 unigenes related to the flooding stress response contained 1,542 SNPs, whereas the 71 contigs involved in the C4 pathway contained 1,827 SNPs. The 100 unigenes related to cellulose biosynthesis accounted for 2,149 SNPs, whereas the 102 contigs annotated as lignin biosynthesis proteins accounted for 2,067 SNPs. Thus, in the present study, there was a larger number of putative SNP markers in the C4 pathway and lignocellulose biosynthesis pathway compared with the results obtained for *P. maximum* [34], in which 1,159 (C4), 491 (cellulose) and 924 (lignin) different SNP positions were identified. Although all of the predicted molecular markers must be validated to eliminate false positives and sequencing errors, the unigene sequences with SNPs that were identified herein provide an important source of data for the study of *U. humidicola*, for which SNPs have not been previously available.

**Table 7:** Summary of the putative SNPs identified using the CLC Genomics Workbench

Number of unigenes	35,093
Total bases	66,854,141
Number of SNPs	560,298
SNP frequency	1 per 119 bp
*Transitions*	358,916
A ↔ G	179,262
C ↔ T	179,654
*Transversions*	192,409
A ↔ C	46,978
A ↔ T	45,773
C ↔ G	52,161
G ↔ T	47,497

### Differences among genotypes

The samples shared a total of 24,057 unigenes, and the BRS Tupi genotype displayed a larger number of unique transcripts (8,585) compared with that of the BH031 genotype (2,419) (Additional file 11). The difference in the quantity of reads between the two genotypes could have influenced the number of unique transcripts.

At the genomic level [12], BH031 is highly genetically divergent from all other *U. humidicola* germplasm accessions. Moreover, five of the 27 (18.5 %) nuclear microsatellite markers that had previously been developed for cv. BRS Tupi did not amplify a product from BH031 [12], and certain SSR markers that had been developed for BH031 did not amplify a product in BRS Tupi [91] (Additional file 12), indicating a different genomic constitution between the two genotypes. A previous study [11] showed that when these genotypes were crossed, the F_1_ hybrids presented several meiotic abnormalities and SSR loci that segregated both in disomy and polysomy, indicating an allopolyploid origin of the species, whereas the different genomic constitutions within the species suggested a hybrid origin of the BH031 genotype.

At the transcript level, significant differences were not observed based on Fisher’s exact test when searching for enriched GO terms among the analyzed BRS Tupi and BH031 plants. We identified 3,202 ORFs that were unique to BRS Tupi, 859 that were unique to BH031 and 18,520 that were shared between the two genotypes. To validate the expression results, three different plants from each genotype were analyzed via qPCR.

### Validation of the differential expression of transcripts among the sexual and apomictic genotypes

Seven reference genes were selected, and primer pairs were designed based on the *U. humidicola* transcriptome sequences (Additional file 13). At the amplification stage, the Uhum_Ea1 and Uhum_18S genes did not amplify or provide different DNA band intensities between the two evaluated genotypes and were discarded. Five primers pairs were then evaluated for qPCR: Uhum_Actin, Uhum_Ubiquitin, Uhum_GAPDH1, Uhum_GAPDH3 and Uhum_Ubiquitin-40S. In the amplification efficiency test, Uhum_GAPDH3 did not provide an E value from 90-110 %. Among the other four primer pairs, the M values were all < 0.5 and the CV values were all < 0.25 (Additional file 14). The Uhum_Ubiquitin and Uhum_Ubiquitin-40s genes presented the lowest M and CV values and were used as reference genes in the present study. Ubiquitin-related genes are usually reported as good reference genes in grasses [92, 93], and the genes selected herein are the first reference genes to be validated for gene expression studies of *U. humidicola* and can be used in other *Urochloa* species (upon validation) as previously described [47] for *U. brizantha*.

Primer pairs were designed for ten differentially expressed transcripts among the two genotypes and ten and eleven BH031 and BRS Tupi unique transcripts, respectively (Additional file 15). All of the primer pairs were evaluated via PCR using genomic DNA as a template. From the differentially expressed category, nine of the ten pairs successfully provided amplicons. Among the ten primer pairs of the BH031 unique transcripts, three primer pairs amplified a product in both genotypes, and among the BRS Tupi unique transcripts, only one primer pair amplified a product in the BH031 genotype (Additional files 16 and 17). Genomic microsatellite primer pairs that were derived from one genotype and analyzed in the other genotype did not amplify a product, indicating that these genotypes were genetically divergent (Additional file 12) as previously observed [12, 91].

After the amplification efficiency analysis of the cDNA, seven primer pairs were selected for the expression analysis: four primer pairs from differentially expressed genes and three primer pairs from unique BH031 transcripts. The evaluated transcripts from the differentially expressed sequences among the analyzed genotypes displayed similarities to the following proteins: triose phosphate/phosphate translocator (TPT; carbon and phosphoenolpyruvate transport [94, 95]), cysteine proteinase 2 (CYSP2; degradation and mobilization of storage proteins in the endosperm [96, 97]), sodium/metabolite cotransporter BASS2 (sodium-dependent pyruvate uptake activity in the plastids [98]) and thiamine thiazole synthase 2 (THI42 [99]). Among the BH031 unique transcripts, two sequences exhibited similarities to premnaspirodiene oxygenase (C7D55; involved in the biosynthesis of solavetivone, a potent antifungal phytoalexin [100]) and calmodulin-binding transcription factor (CMTA1; regulates transcriptional activity in response to calcium signals [101]) proteins, whereas the third sequence showed no similarity to any protein present in the NCBI nr database (no-hit).

**Fig. 7:**
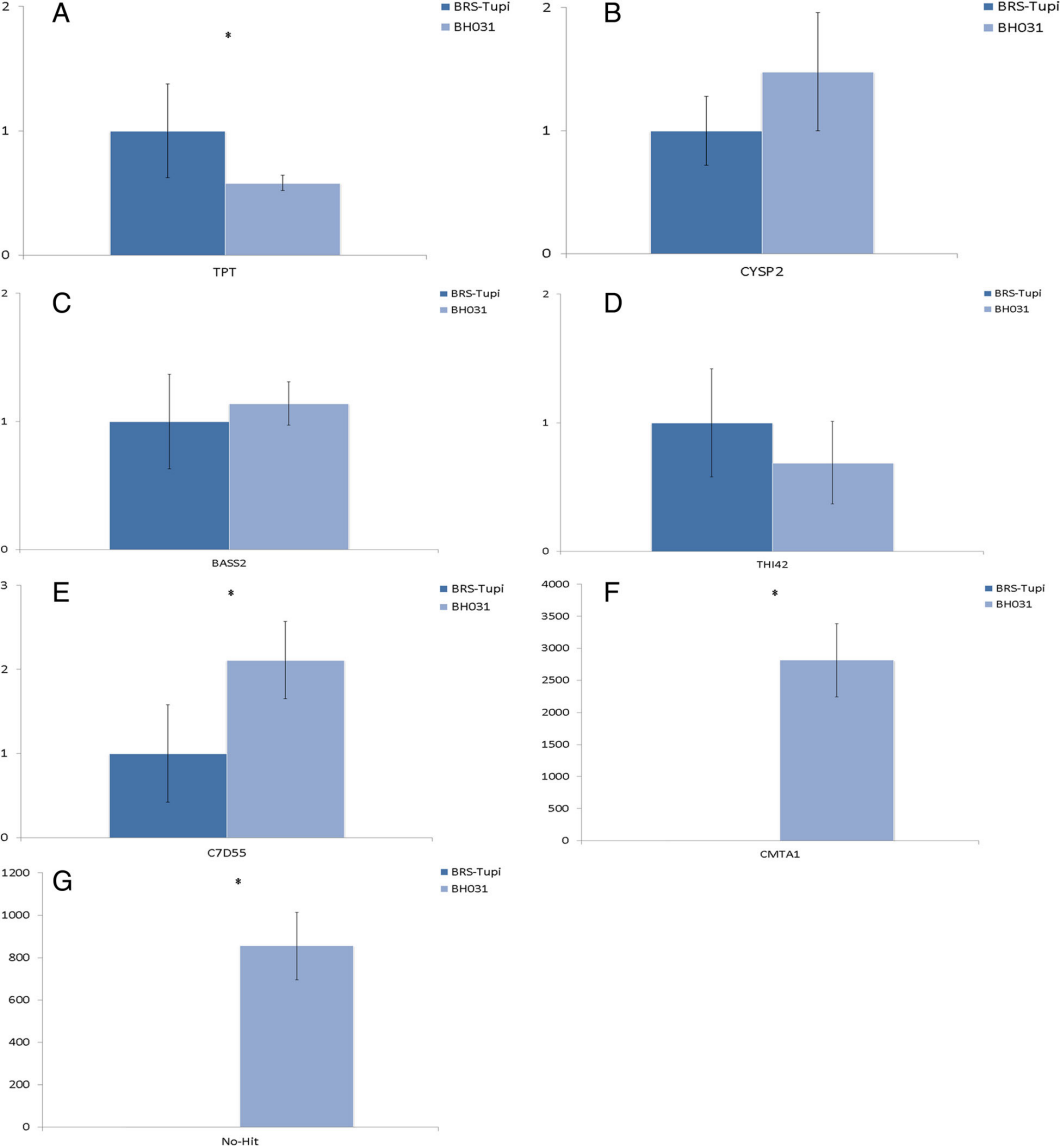
qPCR expression analysis of differentially expressed and unique sequences of BRS Tupi and BH031. **a**: Triose phosphate/phosphate translocator (TPT)*; **b**: cysteine proteinase 2 (CYSP2); **c**: sodium/metabolite cotransporter BASS2; **d**: thiamine thiazole synthase 2 (THI42); **e**: premnaspirodiene oxygenase (C7D55)*; **f**: calmodulin-binding transcription activator (CMTA1)*; **g**: no-hit*. *: Statistically significant differences in gene expression between the genotypes analyzed using REST software

Among the differentially expressed sequences, the TPT transcript displayed the higher difference in expression among the analyzed genotypes; however, the sequence showed greater expression in the BRS-Tupi genotypes (Fig. 7a). Although the CYSP2, BASS2 and THI42 transcripts were more highly expressed in the BH031 genotype in the *in silico* analysis, significant differences were not observed in the expression level of these sequences between BH031 and BRS Tupi plants in the qPCR assays (Figs. 7b, c and d). Among the BH031 unique transcripts, all three transcripts were highly expressed in the three plants (Fig. 7e, f and g). The sequence that was similar to the C7D55 protein presented a 2-fold higher expression level in BH031 plants than in BRS-Tupi plants. The CMTA1 transcript was the most differentially expressed among the genotypes and exhibited nearly 3,000-fold higher expression level in BH031 plants. The no-hit sequence was also highly expressed in the sexual genotypes and showed a 900-fold higher expression level than that in the apomictic genotypes. In general, the results indicated that *in silico* analyses are useful for identifying differentially expressed and unique genes. Certain genes showed divergence among the RT-PCR and quantitative RNA-seq analyses, which could be explained by a lack of sequencing depth and suggests that a new analysis using biological replicates and shorter reads *per* library could provide better results for the *in silico* quantification of mRNA expression.

A transcript that presented similarity to a CMTA protein was apparently silenced in the apomictic genotypes because a DNA coding region was observed but genic expression was not observed; therefore, we decided to verify the presence of other sequences in the transcriptome that showed similarity to CMTA proteins. *CMTA* genes respond differentially and rapidly to hormonal stimuli and environmental stresses [102, 103]. In the family Panicoideae, the number of *CMTA* genes ranges from six (*S. viridis*) to 14 (*P. virgatum*) (Phytozome database, as of June 2016). Another eight transcripts were annotated as CMTA proteins: one for CMTA1, four for CMTA2, two for CMTA4 and one for CMTA6 (Additional file 18). Read mapping statistics showed that two sequences similar to CMTA2 proteins and one sequence similar to CMTA6 proteins had comparable numbers of reads for both genotypes. In contrast, the BRS-Tupi plant presented a larger number of reads for one transcript that presented similarity to a CMTA1 protein and two transcripts that presented similarity to the CMTA4 protein. The sexual genotype BH031 presented a larger number of reads for the other transcripts similar to the CMTA2 protein, and one of these transcripts only presented reads in this genotype.

Our results and the SSR amplification profiles suggest that one of these genotypes likely presents one or more genomic regions that are absent in the other genotype. In addition, certain sequences are silenced in the BRS-Tupi genotypes but transcribed in the BH031 plants. Because the BH031 accession is the only known sexual genotype, we hypothesize that BH031 originated from a cross of *U. humidicola* and an unknown parent. Interspecific hybridization causes considerable changes in gene expression [104], including a loss of paternal imprinting in the hybrids [105]. The BH031 accession would represent a hybrid that has a genome derived from a combination of the genomes of both parents. This scenario would explain the amplification failure of genomic SSRs and transcribed sequences in both genotypes as well as the transcriptional activation of sequences in the BH031 genotype that are silenced in the BRS-Tupi genotype. Further cytogenetic studies are needed to confirm this hypothesis.

### RNA-seq for *U. humidicola* breeding

The domestication and breeding of tropical forage grasses were initiated some decades ago. Similar to other perennial species [55], Koronivia grass remains in the early stages of domestication, which increases the difficulty of identifying concentrated gene groups that play specific roles in important agronomic characteristics. The Koronivia grass breeding program has applied selection methods for many decades, and hybrid progenies were obtained in the early 2000s. Koronivia grass is a perennial crop and necessitate animal trials to release breeding materials, leading to a new cultivar every 10–15 years [1]. In contrast, many agricultural annual crops produce 2–3 generations each year and release cultivars annually.

Next-generation sequencing (NGS)-derived methodologies, such as RNA-seq, have provided rapid advances in genomic data availability. The present study represents an advancement in the description of the *U. humidicola* transcriptome and provides abundant available data via the 2,237 ESTs that were deposited in the NCBI database in March 2015 and more than 35,095 unigenes that were identified via leaf RNA-seq, which included 5,324 unigenes assigned to 327 known metabolic pathways. Moreover, this methodology has facilitated the identification of novel transcripts and new functional markers and improved the SSR database available for this species [11, 22–24].

The high genetic variability available for this species has been demonstrated based on SSR polymorphisms [12]; however, SNP markers are the most abundant type of DNA polymorphism in genomic sequences, and major phenotypic variations have been assigned to this class of markers [106]. Thus, the combination of the RNA-seq approach and SNP identification is ideal for the development of new markers in candidate genes for genetic breeding, and along with conventional breeding, this technique can enhance *U. humidicola* domestication.

## Conclusions

The RNA-seq approach has improved our current knowledge of the transcriptional patterns in the leaves of *U. humidicola* and facilitated the identification of potentially novel genes. The comparison between unigenes that are exclusive to the sexual and apomictic genotypes analyzed herein will allow for the identification and characterization of genes that might be related to aposporic apomixis in this species; however, further evaluation of the reproductive tissues is required. In this study, 4,489 new EST-SSRs and 560,298 new SNP markers that may be associated with important genes for the studied species were identified. Specific SNPs in the sequences of the unigenes involved in the flooding stress response, the C4 pathway and the biosynthesis of cellulose and lignin were identified. The latter could be used to improve forage quality, which is one of the main issues associated with *U. humidicola*. These results provide valuable information related to the genomic resources of *U. humidicola* and other brachiaria grasses for breeding programs. Moreover, important differences between the transcriptomes of the genotypes corroborate genomic observations, which will facilitate investigations of the potential hybrid origin of the sexual BH031 accession. Finally, the *in silico* analysis method was useful for identifying differentially expressed and unique genes in both genotypes.

## Additional files

Additional file 1: Comparison of the *U. humidicola* assembly metrics for the Phytozome transcriptomes. (XLSX 9 kb)
Additional file 2: Species top hits in the NCBI nr database. The five species with greater numbers of top hits in the NCBI nr database compared with the *U. humidicola* unigenes via BLASTX. (PNG 150 kb)
Additional file 3: KEGG glycolysis/gluconeogenesis pathway. The genes that were present in the *U. humidicola* transcriptome are indicated in blue. (PNG 702 kb)
Additional file 4: KEGG plant hormone signal transduction pathway. The genes that were present in the *U. humidicola* transcriptome are indicated in blue. (PNG 771 kb)
Additional file 5: Open reading frames (ORFs) in the *U. humidicola* transcriptome. Description of the types of ORFs identified in the transcript and unigene datasets of the *U. humidicola* transcriptome. (XLSX 9 kb)
Additional file 6: Reciprocal BLAST hit of the assembled unigenes from the *U. humidicola* transcriptome against other grass transcriptomes. Homology search by BLASTn with a cutoff value of 1e-10. (PNG 271 kb)
Additional file 7: List of flooding stress-related genes identified among *U. humidicola* unigenes. (XLSX 9 kb)
Additional file 8: List of genes composing the C4 photosynthetic pathway among the *U. humidicola* unigenes. (XLSX 8 kb)
Additional file 9: List of genes composing the cellulose and lignin pathways among the *U. humidicola* unigenes. (XLSX 10 kb)
Additional file 10: Comparison of the SSRs from *U. humidicola*, *Panicum maximum* and *Brachiaria ruziziensis*. (XLSX 9 kb)
Additional file 11: Venn diagram representing the shared and unique unigenes in the *U. humidicola* transcriptome. (PNG 549 kb)
Additional file 12: Microsatellite amplification profile for the BH031 and cv. BRS Tupi genotypes. Microsatellite amplification profile showing the lack of amplification in the BH031 and cv. BRS Tupi genotypes, which is dependent on the genotype from which the SSR was developed. Loci BhUNICAMP084 (A) and BhUNICAMP065 (B) developed from BH031-enriched libraries [106] and showing the amplification of the SSR markers in BH031 (P1) but not in cv. BRS Tupi (P2); and loci BhUNICAMP082 (C) and BhUNICAMP100 (D) developed from cv. BRS Tupi-enriched libraries [62] and showing the amplification of these SSR markers in cv. BRS Tupi (P2) but not in BH031 (P1). The remaining individuals shown in the polyacrylamide gels are F_1_ hybrids from the cross BH031 x cv. BRS Tupi. (TIF 11136 kb)
Additional file 13: Primer sequences and amplicons of the seven candidate reference genes evaluated in this study. (XLSX 10 kb)
Additional file 14: Gene expression stability values (M) and coefficient of variance (CV) of the candidate reference genes. (XLSX 9 kb)
Additional file 15: Quantitative RT-PCR primer sequences. (XLSX 13 kb)
Additional file 16: Amplification of primer pairs using genomic DNA. BH031 (first sample) and BRS Tupi (second sample). A: Sequences containing reads from both genotypes, B: sequences containing reads from BRS Tupi only, C: sequences containing reads from BH031 only. All of the primer pairs are described in Additional file 15. (PNG 1555 kb)
Additional file 17: Amplification success of the 31 primer pairs for the qPCR assays for both DNA and RNA of genotypes BRS-Tupi and BH031. (XLSX 10 kb)
Additional file 18: Number of reads from the BRS-Tupi and BH031 accessions that presented similarity to CMTA proteins. (XLSX 9 kb)

## Abbreviations

4CL: 4-Hydroxycinnamoyl CoA ligase
ACC synthase: 1-Aminocyclopropane-1-carboxylic acid synthase
ADH: Alcohol dehydrogenase
ART2: Ribosomal RNA transcript antisense to protein 2
BASS2: Sodium/metabolite cotransporter
C3H: P-coumarate 3-hydroxylase
C7D55: Premnaspirodiene
CA: Carbonic anhydrase
CesA: Cellulose synthases
CMTA1: Calmodulin-binding transcription factor
CNPq: Brazilian national council for scientific and technological development
COB: Cobra
COI1: Coronatine Insensitive1
CTL1/POM1: Chitinase-like protein 1
CYP450: Cytochrome P450
CYSP2: Cysteine proteinase 2
EIN2: Ethylene-insensitive protein 2
Embrapa: Brazilian agricultural research Corporation
ERS/ETR1: Ethylene-response sensors
ESTs: Expressed sequence tags
F5H: Ferulate 5-hydroxylase
FAPESP: State of São Paulo Research Foundation
FPKM: Fragments per kilobase of transcript per million mapped reads
G: Coniferyl alcohol (guaiacyl propanol)
GO: Gene ontology
H: p-Coumaryl alcohol (p-hydroxyphenylpropanol)
JA: Jasmonate
JAZ1: Jasmonate ZIM domain-containing protein 1
KAAS: KEGG automatic annotation server
KEGG: Kyoto encyclopedia of genes and genomes database
KO: KEGG orthology
KOB1: Kobito1
KOR: Korrigan
LDH: Lactate dehydrogenase
MISA: MIcroSAtellite script
NAD-ME: NAD-malic enzyme
NADP-ME: NADP-malic enzyme
NCBI: National center of biotechnology information
NGS: Next-generation sequencing
Nr: National center for biotechnology information non-redundant protein database
OLB: Off-Line basecaller software
ORFs: Open reading frames
PCR: Polymerase chain reaction
PDC: Pyruvate decarboxylase
PE: Paired end
PEP: Phosphoenolpyruvate
PEPC: Phosphoenolpyruvate carboxylase
PEPCK: Phosphoenolpyruvate carboxykinase
PPDK: Pyruvate phosphate dikinase
PPR: Pentatricopeptide repeat
psbM: Photosystem II reaction center protein M
qPCR: Quantitative RT-PCR
RBH: Reciprocal BLAST hit
RSEM: RNA-seq by Expectation Maximization
RT-PCR: Reverse transcription polymerase chain reaction
S: Sinapyl alcohol (syringyl propanol)
SAD: Sinapyl alcohol dehydrogenase
SNPs: Single nucleotide polymorphisms
SRA: Short read archive
SSR: Single sequence repeats
SuSy: Sucrose synthase
THI42: Thiamine thiazole synthase 2, chloroplastic
TPT: Triose phosphate/phosphate translocator
Ts: Transition SNPs
Tv: Transversion SNPs
UNICAMP: University of Campinas
