# Supplementary figures and images for: Leaf transcriptome of two highly divergent genotypes of Urochloa humidicola (Poaceae), a tropical polyploid forage grass adapted to acidic soils and temporary flooding areas
Source: BMC Genomics. 2016 Nov 11;17:910. doi: 10.1186/s12864-016-3270-5 (PMC5106776; doi:10.1186/s12864-016-3270-5)

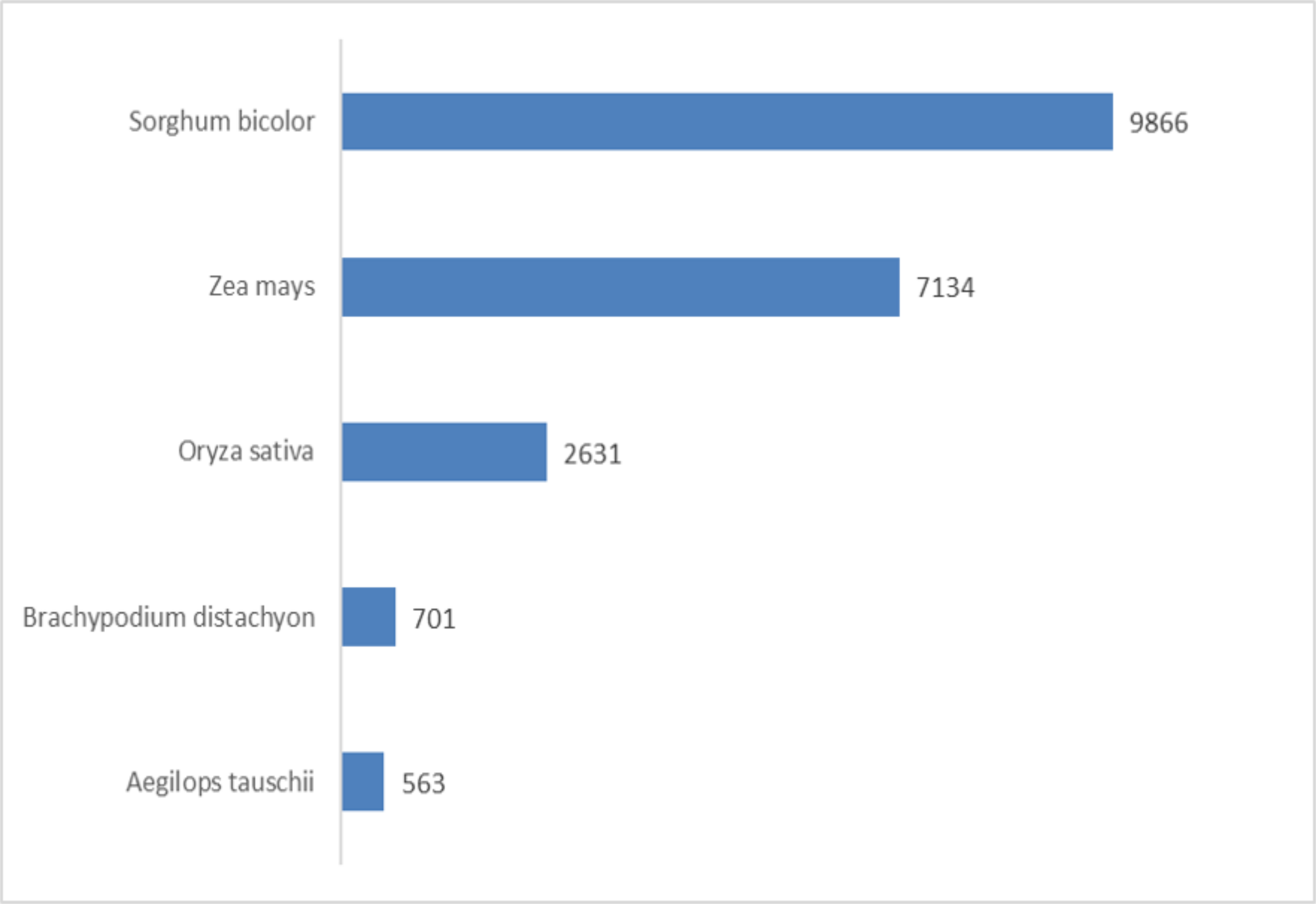

Supplement: Additional file 2: — Species top hits in the NCBI nr database. The five species with greater numbers of top hits in the NCBI nr database compared with the U. humidicola unigenes via BLASTX. (PNG 150 kb) [file 12864_2016_3270_MOESM2_ESM.png]

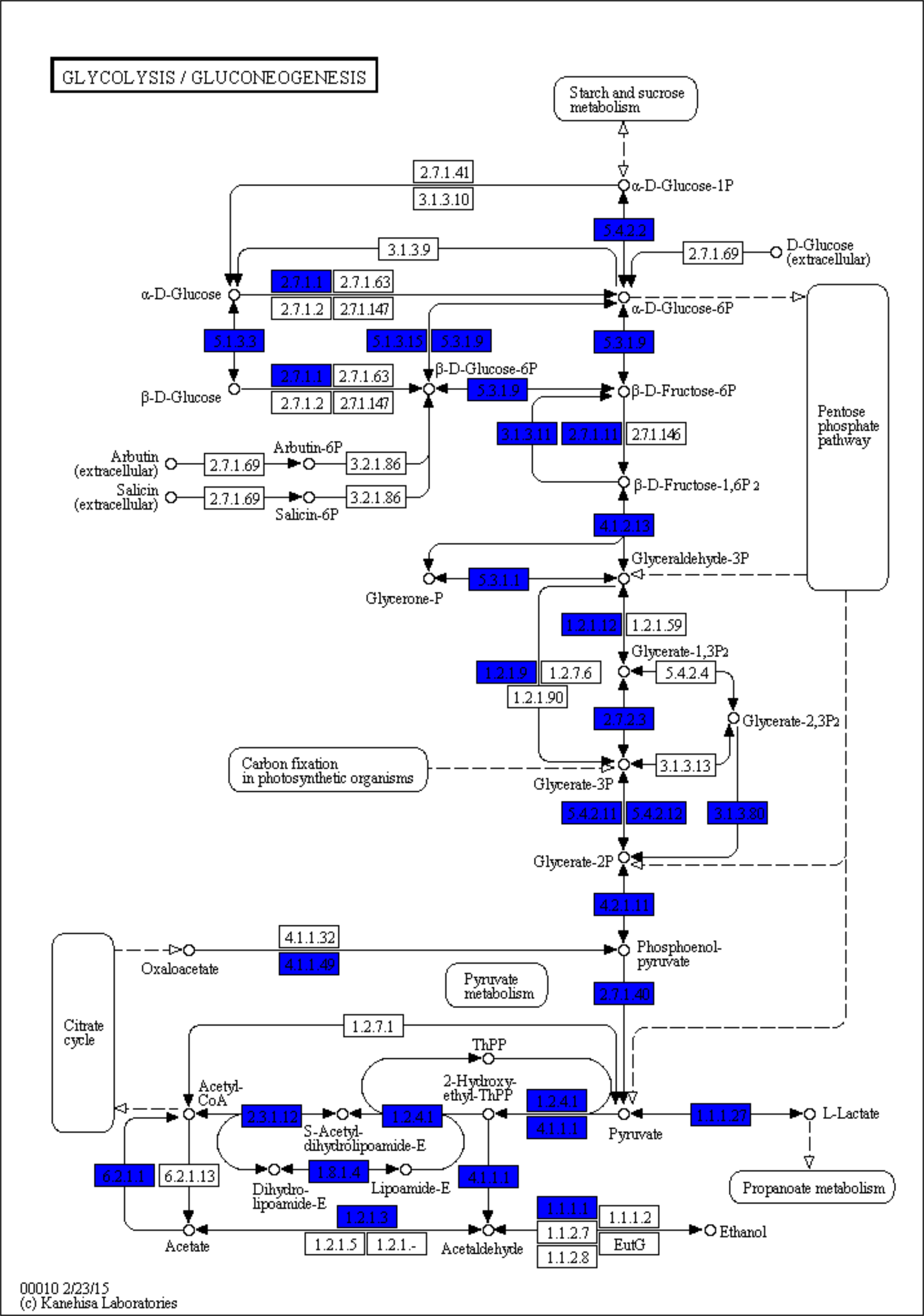

Supplement: Additional file 3: — KEGG glycolysis/gluconeogenesis pathway. The genes that were present in the U. humidicola transcriptome are indicated in blue. (PNG 702 kb) [file 12864_2016_3270_MOESM3_ESM.png]

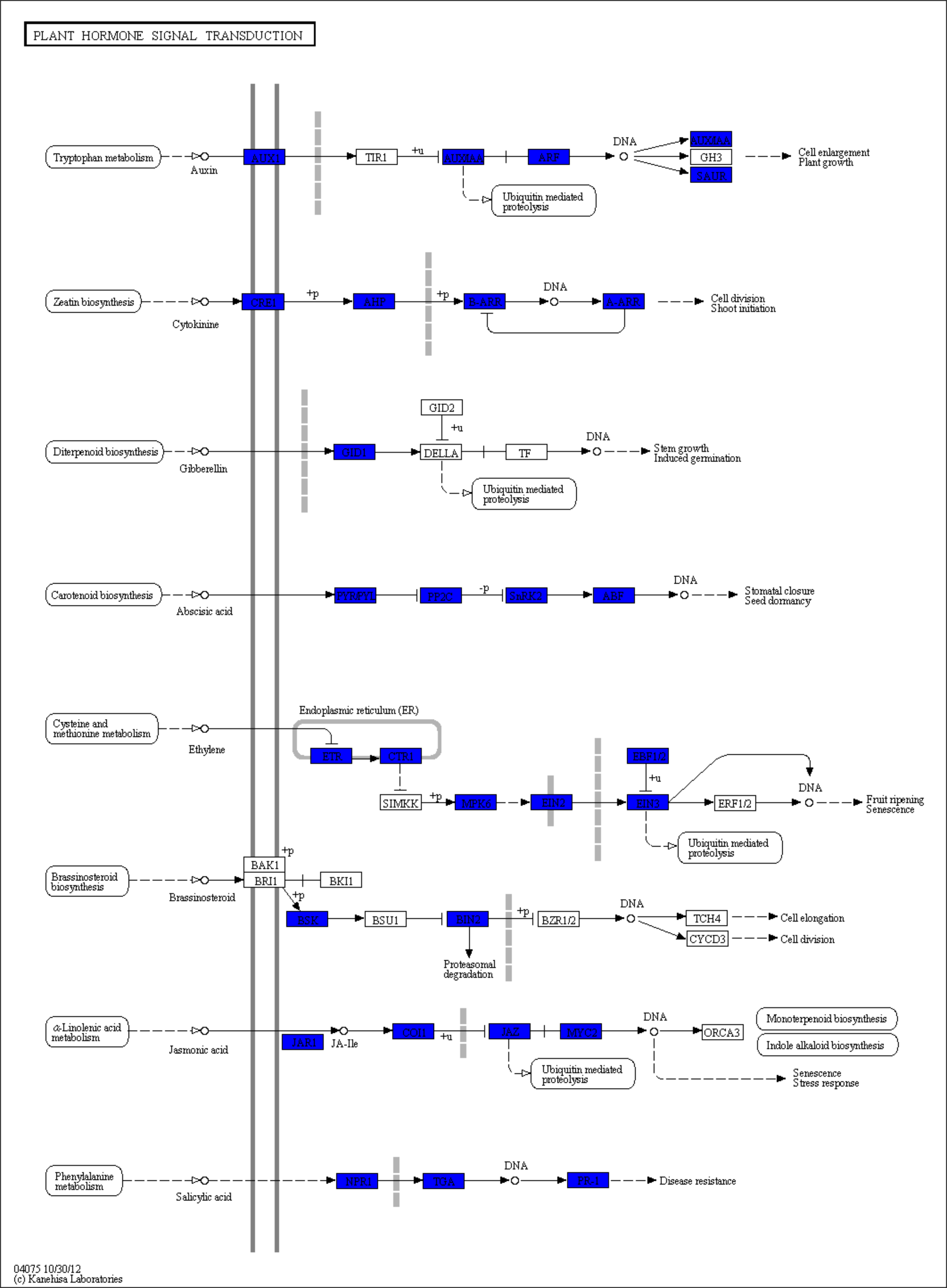

Supplement: Additional file 4: — KEGG plant hormone signal transduction pathway. The genes that were present in the U. humidicola transcriptome are indicated in blue. (PNG 771 kb) [file 12864_2016_3270_MOESM4_ESM.png]

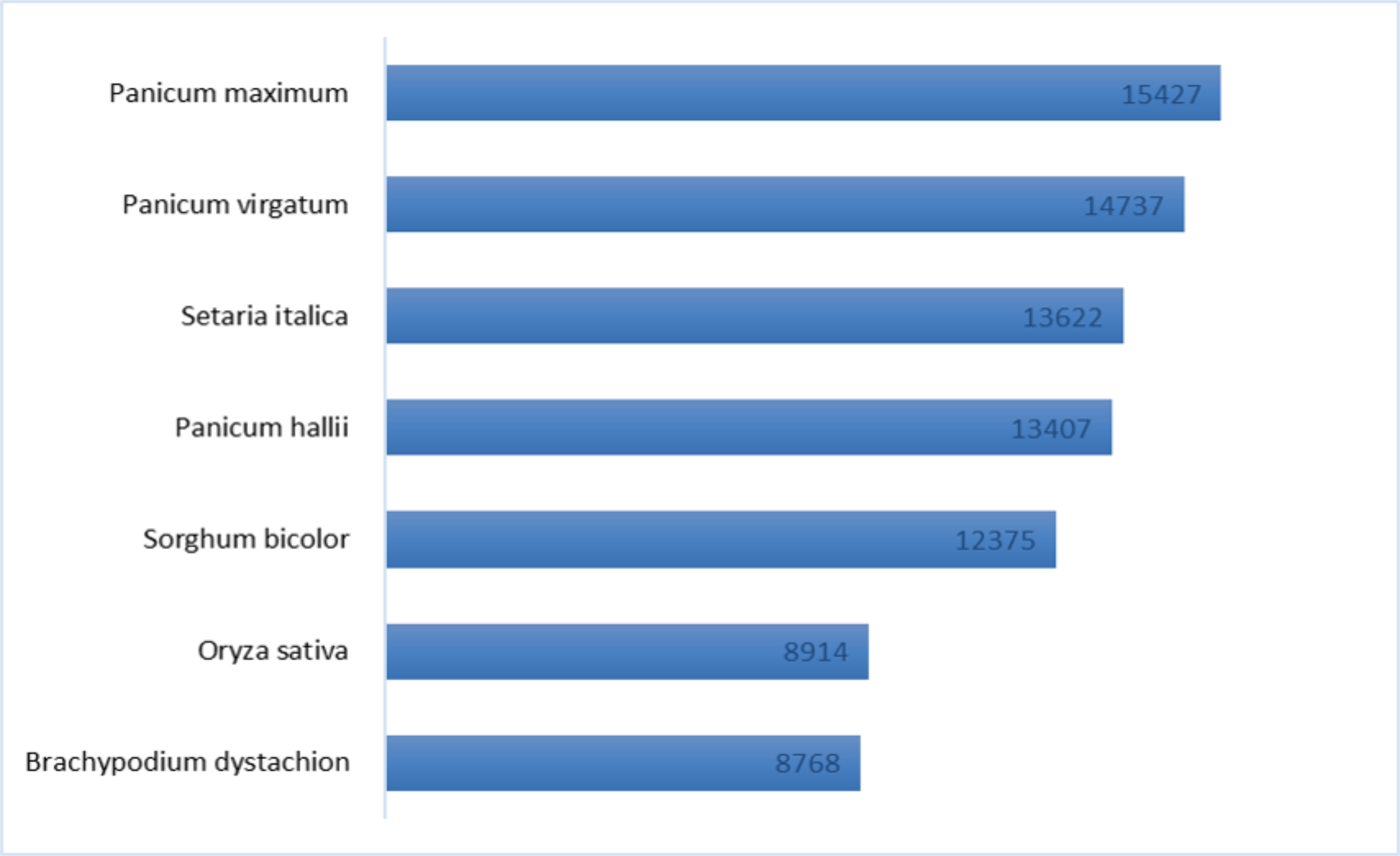

Supplement: Additional file 6: — Reciprocal BLAST hit of the assembled unigenes from the U. humidicola transcriptome against other grass transcriptomes. Homology search by BLASTn with a cutoff value of 1e-10. (PNG 271 kb) [file 12864_2016_3270_MOESM6_ESM.png]

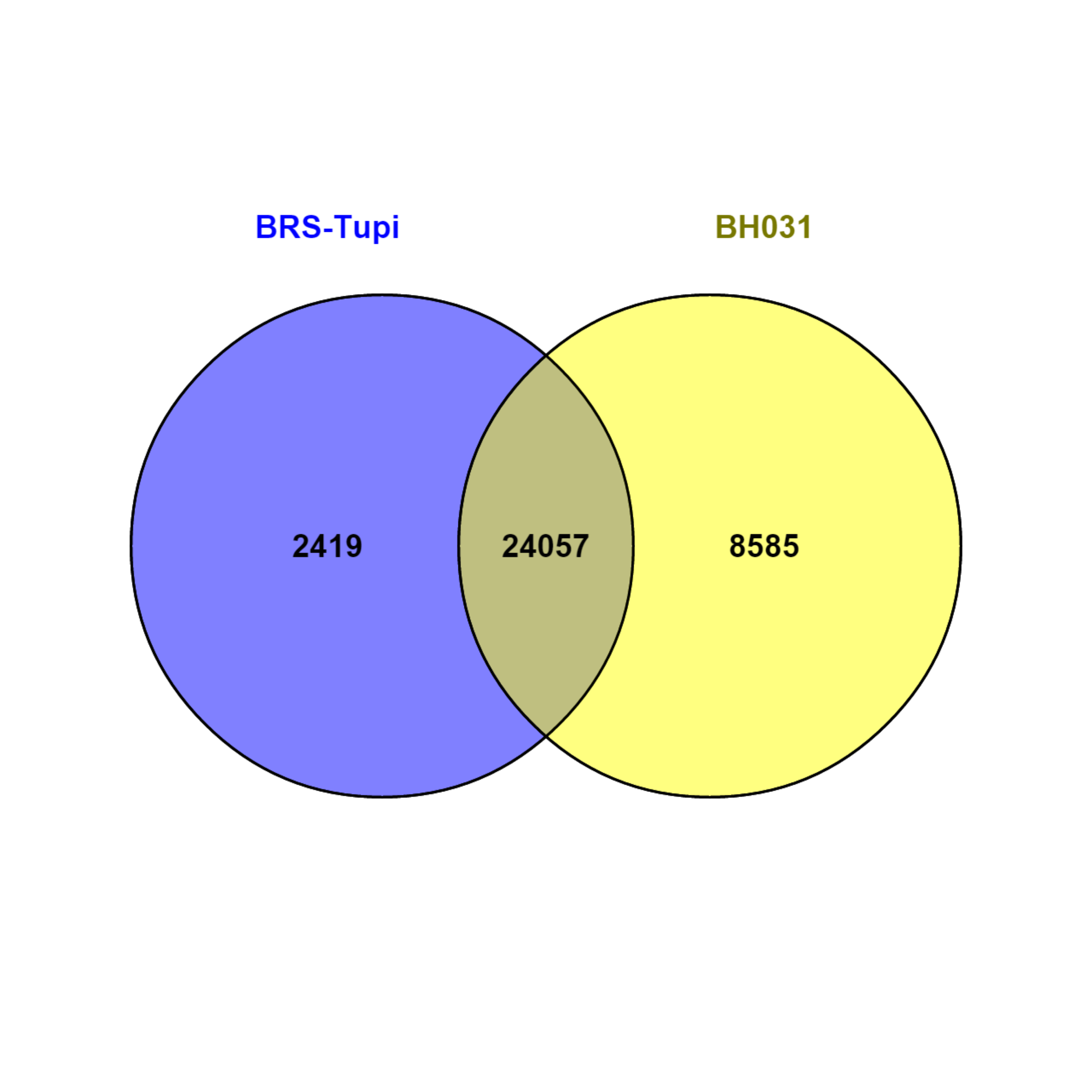

Supplement: Additional file 11: — Venn diagram representing the shared and unique unigenes in the U. humidicola transcriptome. (PNG 549 kb) [file 12864_2016_3270_MOESM11_ESM.png]

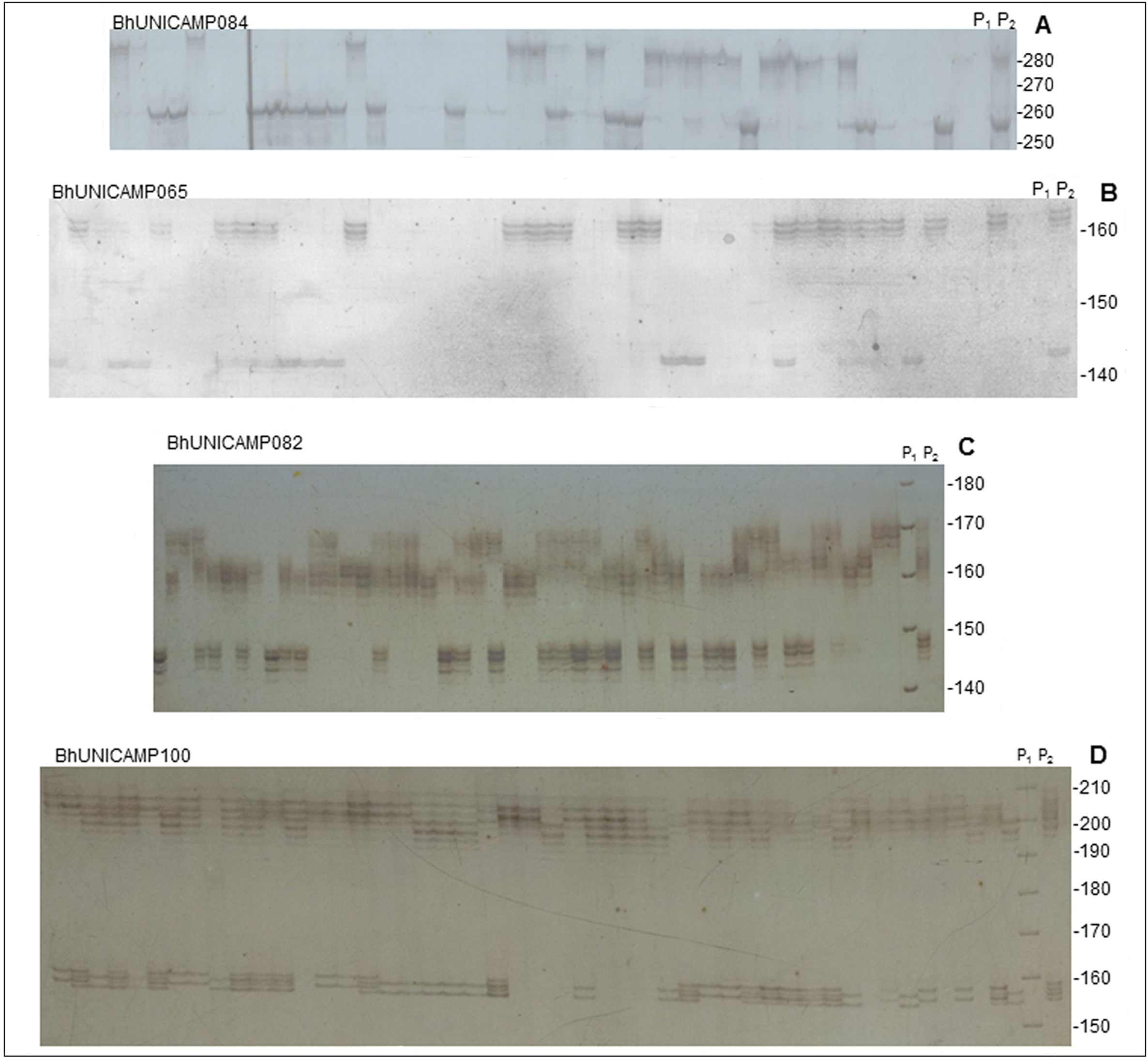

Supplement: Additional file 12: — Microsatellite amplification profile for the BH031 and cv. BRS Tupi genotypes. Microsatellite amplification profile showing the lack of amplification in the BH031 and cv. BRS Tupi genotypes, which is dependent on the genotype from which the SSR was developed. Loci BhUNICAMP084 (A) and BhUNICAMP065 (B) developed from BH031-enriched libraries [106] and showing the amplification of the SSR markers in BH031 (P1) but not in cv. BRS Tupi (P2); and loci BhUNICAMP082 (C) and BhUNICAMP100 (D) developed from cv. BRS Tupi-enriched libraries [62] and showing the amplification of these SSR markers in cv. BRS Tupi (P2) but not in BH031 (P1). The remaining individuals shown in the polyacrylamide gels are F1 hybrids from the cross BH031 x cv. BRS Tupi. (TIF 11136 kb) [file 12864_2016_3270_MOESM12_ESM.tif]

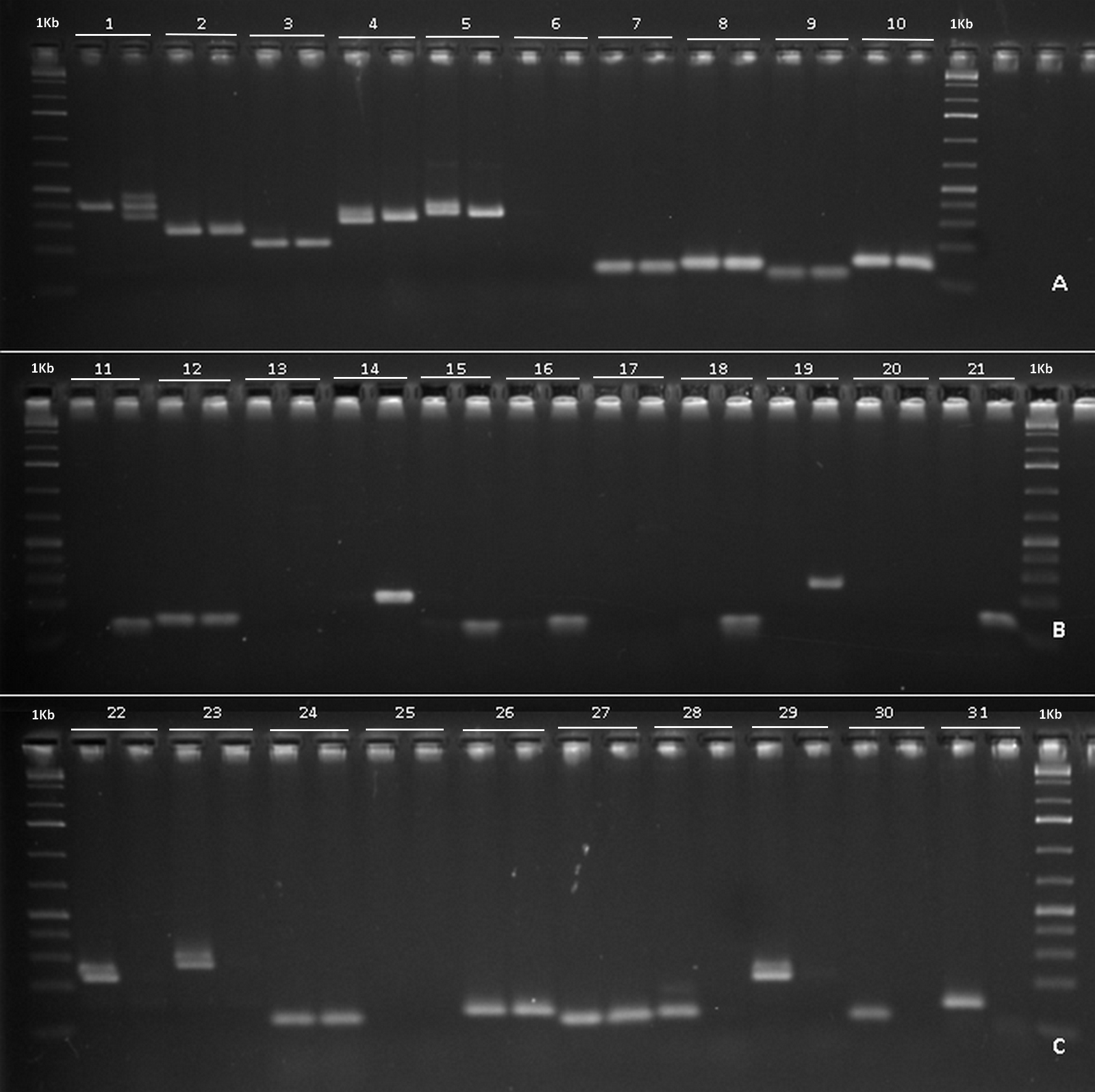

Supplement: Additional file 16: — Amplification of primer pairs using genomic DNA. BH031 (first sample) and BRS Tupi (second sample). A: Sequences containing reads from both genotypes, B: sequences containing reads from BRS Tupi only, C: sequences containing reads from BH031 only. All of the primer pairs are described in Additional file 15. (PNG 1555 kb) [file 12864_2016_3270_MOESM16_ESM.png]
